# Ferroptosis and Alzheimer’s disease: a new insight into neurodegeneration

**DOI:** 10.3389/fimmu.2026.1701767

**Published:** 2026-02-06

**Authors:** Youfang Quan, Hongwei Liu, Minheng Zhang, Meng Li, Bo He, Ke Wang, Xuewen Huang, Shengyang Zhou, Zhongyu Han, Haixia Fan

**Affiliations:** 1 Deyang Hospital Affiliated Hospital of Chengdu University of Traditional Chinese Medicine, Deyang, China; 2 Department of Neurology, Xuanwu Hospital of Capital Medical University, Beijing, China; 3 Department of Neurology, Taiyuan Central Hospital, The Ninth Clinical Medical College of Shanxi Medical University, Taiyuan, Shanxi, China; 4 Department of Gerontology, The First People’s Hospital of Jinzhong, Shanxi, China; 5 School of Medical and Life Sciences, Chengdu University of Traditional Chinese Medicine, Chengdu, China; 6 Zhongda Hospital, Southeast University, Nanjing, China; 7 Department of Sleep Center, First Hospital of Shanxi Medical University, Taiyuan, Shanxi, China

**Keywords:** AD, Aβ, ferroptosis, inflammation, microglia, neuroinflammation, NFTs

## Abstract

Alzheimer’s disease (AD), a chronic and progressive neurodegenerative disorder, poses a significant threat to the health of the aging population. The pathological hallmarks of AD include the accumulation of amyloid-β (Aβ) plaques and neurofibrillary tangles (NFTs) within the brain. While substantial neuronal loss has been consistently observed in AD, the precise mechanisms underlying neuronal elimination remain incompletely understood. As a distinct form of regulated cell death, the contribution of ferroptosis to AD pathogenesis and progression warrants further investigation. This review critically examines the amyloid cascade hypothesis within the context of AD, with particular emphasis on the molecular signatures of ferroptosis and their contributions to canonical AD pathogenesis and cognitive decline. We aim to provide an updated perspective on AD etiopathogenesis. Furthermore, we synthesize current therapeutic strategies targeting ferroptosis inhibition in AD, highlighting recent advances that hold significant implications for guiding present and future translational efforts.

## 1 Introduction

Alzheimer’s disease (AD) is a neurodegenerative disorder characterized by progressive cognitive decline, memory loss, and neuropsychiatric symptoms, representing one of the most critical global public health challenges of the 21st century (1). Currently, it is estimated that over 50 million individuals worldwide are living with AD, and this number is projected to triple by 2050, imposing a profound socioeconomic burden (2, 3). Despite more than a century of dedicated research, the core pathogenic mechanisms of AD remain incompletely understood. Current diagnosis still primarily relies on two canonical neuropathological hallmarks: extracellular Aβ plaques and intracellular neurofibrillary tangles composed of hyperphosphorylated Tau protein (4, 5). However, therapeutic development targeting these pathways has faced repeated setbacks. Most clinical trials, including those involving the conditionally approved agent aducanumab, have failed to demonstrate unequivocal clinical benefit (6, 7). Although lecanemab has shown efficacy in slowing cognitive decline in early AD in clinical trials, its long-term safety, broad clinical relevance, and therapeutic accessibility require further validation (8, 9). These persistent challenges underscore the limitations of the traditional “amyloid cascade hypothesis” and necessitate a paradigm shift—from a single-protein-centric perspective towards a more systemic and integrated molecular network approach—to unravel the pathogenic origins of AD.

In this context, ferroptosis—a newly defined, regulated cell death modality driven by iron-dependent lipid peroxidation—has rapidly emerged as a frontier and focus in AD research (10, 11). This process is centrally characterized by intracellular iron overload that initiates the Fenton reaction, catalyzing massive reactive oxygen species (ROS) generation; this in turn triggers the accumulation of lipid peroxidation products (e.g., 4-hydroxynonenal), protein oxidative damage (including promotion of Aβ1–42 aggregation), and DNA strand breaks (12). Concurrently, glutathione (GSH) depletion coupled with functional inactivation of glutathione peroxidase 4 (GPX4) collapses the cellular antioxidant defense system. Morphologically, ferroptosis is distinguished from other cell death modalities by characteristic ultrastructural alterations such as mitochondrial cristae dissolution and outer membrane condensation (13, 14).

**Figure 1:**
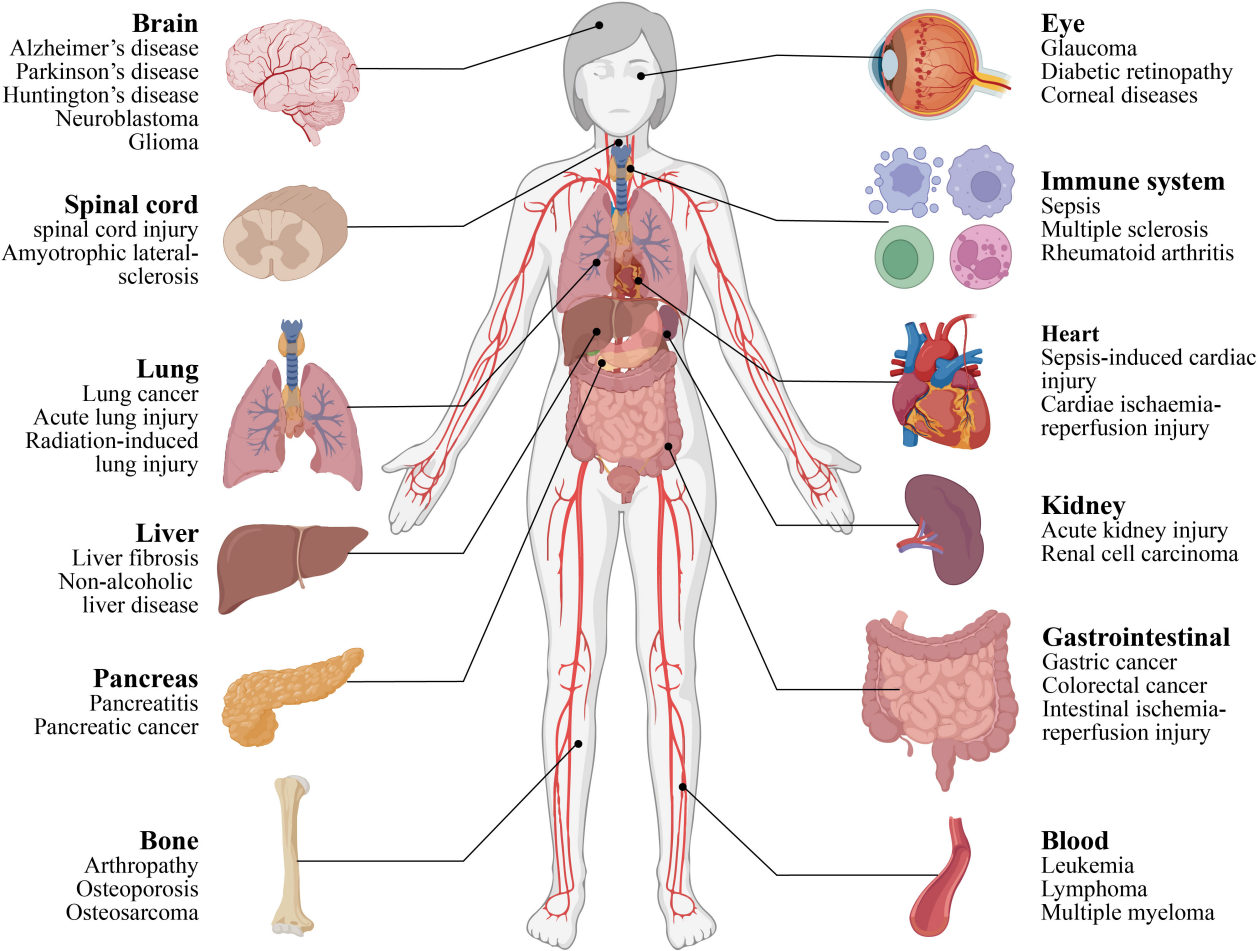
Ferroptosis is associated with diseases of various tissues and organs throughout the body. This figure provides a panoramic overview of major human organ systems and their associated prototypical diseases, thereby contextualizing the broad implications of ferroptosis across a spectrum of multisystem disorders. Centered on the central nervous system, the brain (depicted on the left) is associated with neurodegenerative conditions including AD and Parkinson’s disease, which directly aligns with the established pathological role of ferroptosis in AD. The figure further encompasses organs such as the spinal cord, lungs, liver, pancreas, and bones, alongside systems including the eyes, immune system, heart, kidneys, gastrointestinal tract, and hematopoietic system, each paired with their clinically relevant disorders. Collectively, this illustration underscores ferroptosis as a conserved, regulated cell death pathway that contributes to the pathogenesis of diseases spanning multiple organ systems.

Ferroptosis, a regulated form of cell death, plays a significant role in various pathological conditions, including neurodegeneration, cancer, and ischemia–reperfusion injury (**Figure 1**) (15–17). Its connection to AD was first established through key pathological evidence: Sayre et al. demonstrated that transition metals such as iron and copper bound within Aβ plaques and neurofibrillary tangles contribute substantially to oxidative catalytic activity, laying the foundation for understanding metal-driven oxidative stress and ferroptosis in AD (18). Subsequent studies further revealed a stage-specific, reciprocal network linking iron dysregulation, ferroptosis, Aβ/tau pathology, and neuroinflammation—three core components of AD pathogenesis. To clarify the causal relationships and stage−dependent roles of these factors, we propose a conceptual framework for AD progression (1):Early AD (causal events): Disruption of cerebral iron homeostasis leads to regional iron accumulation, which primes neurons for ferroptosis through elevated ROS and impaired antioxidant defenses (2).Transitional AD (facilitative events): Ferroptotic stress promotes Aβ aggregation and tau hyperphosphorylation. In turn, Aβ enhances iron uptake via transferrin receptor 1, and phosphorylated tau suppresses GPX4 expression, forming a self−reinforcing loop. Concurrently, ferroptotic death releases DAMPs, activating glial cells and initiating neuroinflammation, which further impairs iron homeostasis and antioxidant capacity (3).Late AD (downstream events): Sustained ferroptosis contributes to widespread neuronal loss, while progressive Aβ/tau deposition and chronic neuroinflammation drive synaptic dysfunction, circuit disruption, and clinical cognitive decline (19).

This review aims to systematically elucidate the central role of iron dyshomeostasis and the ferroptosis process it drives in the pathogenesis of Alzheimer’s disease. Based on an integrated analysis of evidence from post-mortem pathology, neuroimaging, and experimental models, we propose that neuronal ferroptosis triggered by cerebral iron overload may constitute an early critical event that initiates and propels the subsequent pathological cascade in AD. This article provides an in-depth analysis of the molecular mechanisms underlying ferroptosis and delineates its complex interactions with the core pathological hallmarks of AD. We advocate that future AD research must establish a comprehensive framework that integrates iron metabolism, oxidative stress, and Aβ/tau pathology to transcend the limitations of the traditional amyloid-centric hypothesis, thereby achieving a more holistic understanding of AD pathogenesis. Furthermore, this review critically evaluates current therapeutic strategies targeting the ferroptosis pathway, with the objective of providing a theoretical foundation for developing novel interventional approaches.

## 2 AD

In the early 20th century, German psychiatrist Alois Alzheimer, using silver-staining techniques on the brain tissue of a patient with cognitive decline, first described two hallmark lesions: extracellular senile plaques composed of β-amyloid deposits and intraneuronal neurofibrillary tangles (20). This discovery challenged the prevailing view of dementia as an inevitable consequence of aging. In 1910, his mentor, Emil Kraepelin, named the condition “Alzheimer’s disease,” emphasizing its distinct pathological nature independent of age. Based on the age of onset, AD is classified into early-onset AD (EOAD, <65 years) and late-onset AD (LOAD, ≥65 years). LOAD is predominantly associated with sporadic risk alleles, exhibiting both genetic and etiological heterogeneity, where genetic and environmental factors collectively modulate disease risk and progression rate. Among these, the APOE ϵ4 allele represents the most well-established and robust genetic risk factor for LOAD, significantly increasing disease susceptibility in carriers (21–23).

The traditional “amyloid cascade hypothesis” posits that Aβ oligomers are the central pathogenic initiators in AD. They are proposed to trigger the disease process by activating glial cells, impairing synaptic plasticity, and inducing neuronal toxicity. Subsequently, abnormal Tau phosphorylation further disrupts axonal transport, ultimately leading to neuronal death. Nevertheless, some clinical-pathological studies(postmortem brain tissue samples from AD patients) has revealed significant limitations in this theoretical framework (24). For instance, transgenic mice overexpressing APP develop Aβ deposits but fail to form typical neurofibrillary tangles or exhibit significant neuronal loss (25). Concurrently, clinical trials targeting Aβ or Tau (e.g., using aducanumab or gosuranemab) have yielded only limited cognitive benefits, falling short of expectations and further highlighting the inadequacy of the traditional hypothesis (26, 27). These findings collectively indicate that focusing solely on Aβ toxicity is insufficient to fully explain the mechanisms underlying neurodegeneration.

Given these limitations, the establishment of a conceptual model capable of integrating multiple pathological processes and elucidating their spatiotemporal dynamics becomes imperative. We propose an integrative framework centered on the “iron dyshomeostasis-ferroptosis” axis, aiming to transcend the traditional linear cascade hypothesis. This model posits that during the early stages of AD, particularly in the preclinical phase, cerebral iron overload—triggered by genetic factors (e.g., APOE ϵ4) or aging—along with the resultant oxidative stress, constitutes a key upstream event that initiates the pathological cascade. The interactions between ferroptosis and Aβ/Tau pathologies, along with its amplifying effects on neuroinflammation, will be elaborated in subsequent chapters.

In the late stages of the disease, these interconnected pathways—ferroptosis, Aβ deposition, Tau tangle formation, and chronic neuroinflammation—converge and synergize, culminating in extensive neuronal loss and synaptic failure. Within this framework, ferroptosis is not merely a downstream consequence of Aβ/Tau pathology but also an upstream driver and a sustained propagator throughout the disease continuum. This model provides a more systematic and dynamic perspective for understanding the progression of AD from a compensable early phase to an irreversible advanced stage, thereby laying a theoretical foundation for developing precision combination therapies tailored to different disease stages.

## 3 The molecular mechanisms of ferroptosis

### 3.1 Iron metabolism and ferroptosis

**Figure 2:**
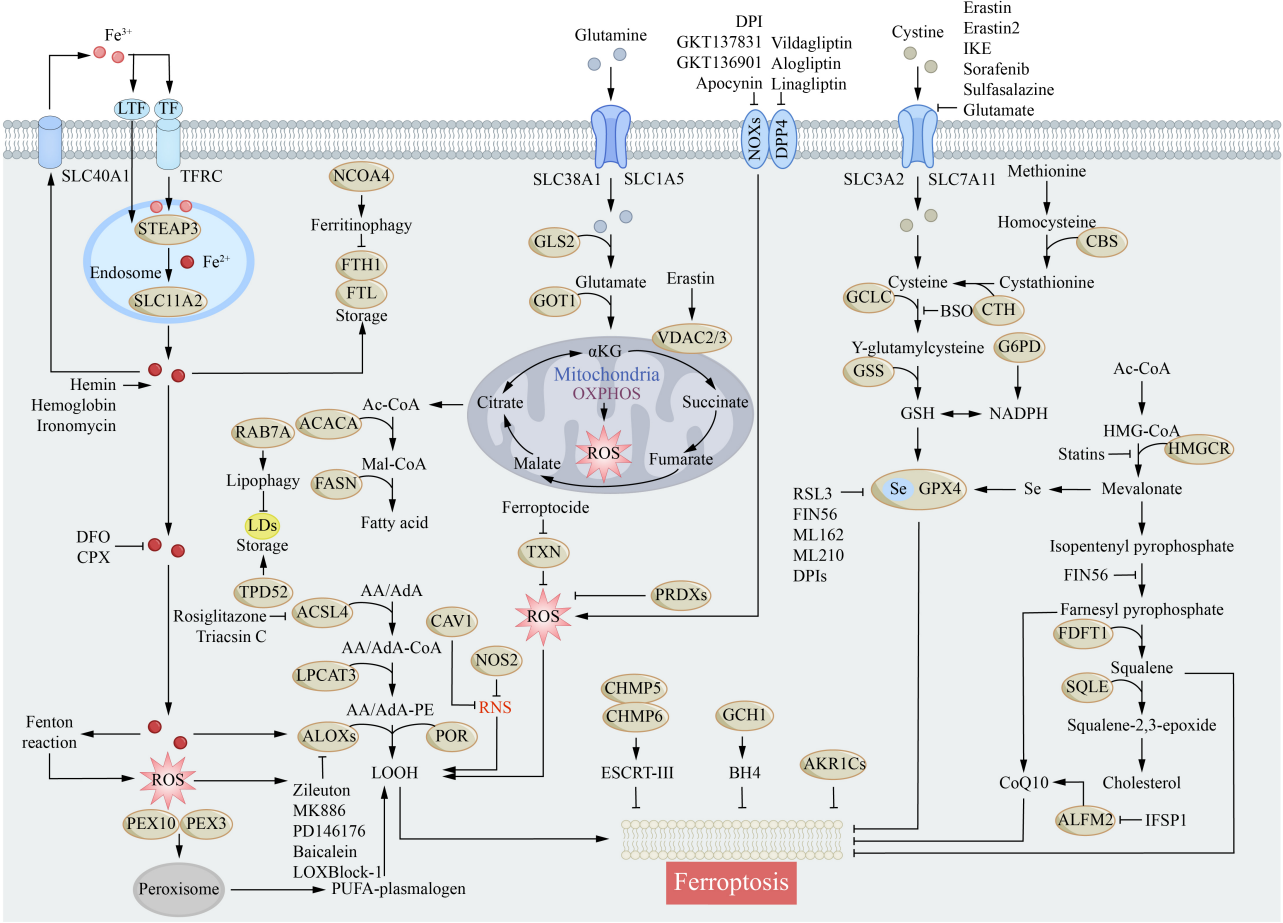
Molecular mechanisms of ferroptosis. Ferroptosis is primarily induced by iron-mediated lipid peroxidation. This schematic delineates the core molecular circuitry of ferroptosis, a regulated iron-dependent lipid peroxidation-driven cell death pathway, and its direct implications for AD pathogenesis. It maps three interconnected axes of ferroptosis (1): Iron dyshomeostasis, where extracellular iron import via TFRC, ferritinophagy, and lipophagy elevate labile iron pools, fueling Fenton reaction-mediated ROS production— a process amplified in AD by Aβ and tau-induced iron accumulation in neurons and glia (2). Lipid peroxidation cascades, in which ACSL4/LPCAT3-mediated PUFA phospholipid synthesis and ALOX/POR-driven oxidation generate toxic lipid hydroperoxides (LOOH), damaging neuronal membranes and accelerating Aβ/tau aggregation in AD (3). Antioxidant system collapse, where impaired cystine import (via SLC7A11/SLC3A2) and GSH depletion compromise GPX4-dependent peroxide detoxification— a critical vulnerability in AD brains.

Iron homeostasis is maintained by a sophisticated network of absorption, transport, storage, and efflux (28, 29). Dysregulation of iron metabolism, particularly impaired Tf/TfR1 uptake leading to an elevated labile iron pool, constitutes a core mechanism of ferroptosis (**Figure 2**) (30, 31). Brain iron is primarily acquired via transferrin-bound iron and non-transferrin-bound iron pathways (32, 33). TfR1 mediates efficient iron transport across the blood-brain barrier, with distinct neural cell types employing specific uptake mechanisms. Neurons and microglia rely predominantly on the Tf/TfR1 pathway and DMT1-mediated NTBI uptake; oligodendrocytes utilize the H-ferritin receptor; and astrocytes employ a multifaceted strategy including Tf/TfR1, DMT1, and perivascular uptake (34, 35). Systemic iron homeostasis is regulated by hepcidin, which degrades ferroportin, the primary cellular iron exporter, thereby preventing oxidative damage from free iron (36, 37). Disruption of this homeostatic network, especially during critical developmental periods, can lead to persistent cognitive deficits (38, 39).

Ferroportin 1 (Fpn), the sole known mammalian non-heme iron exporter, is widely distributed in neurons, astrocytes, oligodendrocytes, and brain microvascular endothelial cells and is crucial for murine embryonic development, forebrain patterning, and neural tube closure (40, 41). Studies indicate that Fpn expression is downregulated in AD mouse models and in the brains of AD patients (42, 43). Neuron-specific knockout of Fpn in the cortex and hippocampus induces AD-like hippocampal atrophy and memory impairment in mice (44). Subsequent rescue experiments have demonstrated that restoring Fpn expression ameliorates both ferroptosis pathology and memory function. Notably, both Fpn-knockout mice and AD model mice exhibit classic morphological and molecular hallmarks of ferroptosis. Gene set enrichment analysis of ferroptosis-related transcriptomic data further reveals that differentially expressed genes are significantly enriched in AD-associated gene sets (44). Intriguingly, Fpn mRNA levels remain unchanged in some AD mouse models, suggesting potential post-transcriptional dysregulation of Fpn. Collectively, these findings establish that Fpn dysregulation and subsequent ferroptosis play an important role in AD progression, offering novel targeted therapeutic strategies for the disease.

### 3.2 Lipid peroxidation in ferroptosis

A defining hallmark of ferroptosis involves the accumulation of lipid peroxidation, primarily driven by oxidative damage to polyunsaturated fatty acid-containing phospholipids (PUFA-PLs) (45, 46). The molecular structure of PUFAs, featuring multiple oxidatively vulnerable double bonds in their carbon chains, renders them particularly susceptible to peroxidation within membrane phospholipids such as phosphatidylethanolamine (PE) and phosphatidylinositol (PI), establishing these lipids as primary targets (47, 48). Key regulatory roles in ferroptosis are played by acyl-CoA synthetase long-chain family member 4 (ACSL4) and lysophosphatidylcholine acyltransferase 3 (LPCAT3). ACSL4 specifically activates long-chain PUFAs, including arachidonic acid (AA) and adrenic acid (AdA), via conjugation to coenzyme A. LPCAT3 subsequently esterifies these activated PUFAs into membrane phospholipids, generating peroxidation-susceptible lipid pools such as PE-AA and PE-AdA (47, 48). Inhibition of this enzymatic cascade significantly reduces lipid peroxide accumulation, effectively blocking ferroptosis progression (49). Notably, ferroptosis induction strictly depends on PUFA incorporation into phospholipids, as free oxidized PUFAs lack cell death-inducing capacity (50). Related *in vitro* and *in vivo* (HeLa cell line and mouse heterotopic tumor model) research identifies protein kinase C βII (PKCβII) as a crucial molecular sensor of lipid peroxidation; it detects initial lipid peroxidation signals and amplifies the peroxidation cascade through ACSL4 phosphorylation, thereby positively regulating ferroptosis (51). These findings establish ACSL4 as a novel ferroptosis regulator and reveal promising therapeutic targets for ferroptosis-related pathologies.

APOE gene polymorphism (ϵ2/ϵ3/ϵ4) profoundly influences ferroptosis susceptibility and neurodegenerative progression by remodeling neural lipid metabolism networks. The high-risk APOEϵ4 allele impairs ABCA1-mediated cholesterol efflux, disrupting neuronal membrane cholesterol/phospholipid ratios and promoting abnormal accumulation of PUFA-enriched phosphatidylethanolamine (PUFA-PE) in the plasma membrane. These phospholipids readily undergo Fe²^+^-dependent lipid peroxidation, creating a pro-ferroptotic microenvironment (52, 53). Concurrently, APOEϵ4 downregulates SLC7A11 expression, limiting cystine uptake and further compromising GPX4-mediated antioxidant defenses. In contrast, the APOEϵ2 allele activates the Nrf2 pathway, inducing expression of FSP1 and GSH synthetase to reconstitute the coenzyme Q10-vitamin E antioxidant axis, thereby markedly reducing PUFA-PE peroxidation susceptibility (54). This genotype-dependent modulation of lipid metabolism not only underscores the critical role of APOE polymorphism in ferroptosis regulation but also provides molecular insights into differential neurodegenerative disease susceptibility across genotypes.

4-Hydroxynonenal (4-HNE), a characteristic end-product of ω-6 polyunsaturated fatty acid (e.g., arachidonic acid) lipid peroxidation, serves as a key molecular biomarker of ferroptosis (55). This highly reactive aldehyde contains strongly electrophilic carbonyl groups that selectively modify sulfhydryl and amino groups within presynaptic membrane proteins (such as synaptophysin and vesicular glutamate transporters), disrupting synaptic vesicle docking and exocytosis. Such modifications lead to aberrant neurotransmitter release and ultimately trigger cellular ferroptosis (56, 57). Beyond its direct cytotoxic effects during ferroptosis, 4-HNE may indirectly promote cell death by modulating intracellular signaling pathways or impairing mitochondrial function. Furthermore, 4-HNE readily forms covalent adducts with Aβ peptides through cross-linking reactions, accelerating amyloid fibril formation and pathological deposition – a process critically implicated in the pathogenesis of neurodegenerative disorders including AD (58).

Cumulatively, the core mechanism of ferroptosis involves synergistic amplification of lipid peroxidation cascades through multiple feedback loops. This process extends beyond mere accumulation of lipid peroxides, engaging a complex pathological network modulated by diverse regulatory factors. These insights advance our mechanistic understanding of ferroptosis and illuminate its potential connections to neurodegenerative pathologies. Future therapeutic strategies targeting ferroptosis-associated pathways may reveal promising avenues for treating neurodegenerative diseases.

### 3.3 Antioxidant defense systems in ferroptosis

#### 3.3.1 GPX4/GSH axis

GPX4 serves as the central executor of ferroptosis defense, and its function relies on the cysteine-glutathione metabolic axis mediated by SLC7A11. The System Xc^-^ transporter composed of SLC7A11 imports extracellular cystine into cells while exporting glutamate through an antiport mechanism. This process is a crucial prerequisite for GSH biosynthesis and also an important molecular switch for ferroptosis initiation (59). The xCT subunit with its 12 transmembrane domains mediates substrate transport, while the single-pass 4F2hc subunit ensures membrane localization and complex stability (60). Widely distributed in the central nervous system, this antiporter exports glutamate and imports cystine (Cys_2_) (61, 62). This exchange process represents a significant molecular trigger for ferroptosis initiation (45).

Intracellular cystine is reduced to cysteine, the key precursor for GSH synthesis, while NADPH from the pentose phosphate pathway supplies reducing equivalents for this antioxidant system. Through its thiol group (–SH), GSH neutralizes lipid peroxides and thus inhibits ferroptosis (63). Under pathological conditions, impaired cystine uptake depletes GSH, depriving GPX4 of essential reducing support and ultimately inducing ferroptosis. Therefore, GPX4 expression and activity can serve as sensitive biomarkers for monitoring ferroptosis progression (64).

Unlike antioxidant enzymes such as SOD and CAT that scavenge free ROS, GPX4 specifically targets lipid peroxidation products in the phospholipid bilayer of cell membranes (65). By consuming GSH, GPX4 reduces cytotoxic phospholipid hydroperoxides (PLOOH) to harmless phospholipid alcohols (PLOH), thereby blocking the lipid peroxidation chain reaction and maintaining membrane integrity (66).

This axis is closely associated with the occurrence and development of AD: there is a significant increase in oxidative stress and a decrease in GSH levels in the AD brain, leading to impaired GPX4 activity, massive accumulation of lipid peroxidation products in neuronal cell membranes, and accelerated neuronal ferroptosis. In addition, Aβ deposition and excessive tau phosphorylation in the brains of AD patients can further inhibit the expression of SLC7A11, exacerbating GSH depletion and GPX4 inactivation, and forming a vicious cycle of “oxidative stress-ferroptosis-AD pathological damage”. Targeted enhancement of GPX4 activity or restoration of GSH synthesis capacity can effectively reduce neuronal ferroptosis in the brains of AD model animals and improve cognitive dysfunction, suggesting that this axis is a potential therapeutic target for AD.

Through this specialized mechanism, GPX4 consumes GSH to catalytically reduce cytotoxic membrane phospholipid hydroperoxides (PLOOH) into harmless phospholipid alcohols (PLOH), directly interrupting the lipid peroxidation chain reaction and preserving membrane integrity. This catalytic process critically depends on the selenocysteine (Sec) residue at its active site, with selenium deficiency significantly impairing GPX4 enzymatic activity (67). As previously established, the cystine-GSH metabolic axis supplies essential reducing equivalents for GPX4 function, where system Xc^-^-mediated cystine uptake represents the rate-limiting step for GSH biosynthesis. Pharmacological inhibition of this pathway by agents such as erastin depletes GSH reserves, directly inactivating GPX4 through loss of reducing power and culminating in uncontrolled membrane lipid peroxidation (68).

#### 3.3.2 Nrf2/ARE axis

Transcription factor Nrf2 is a core regulator of cellular oxidative stress responses. It drives the expression of downstream antioxidant genes by activating the ARE, thereby regulating antioxidant defense and ferroptosis processes (63). Nrf2’s functional coordination is achieved through its domains: C-terminal Neh1 and Neh3 mediate DNA binding and transactivation, respectively, while N-terminal Neh2 enables Keap1-dependent negative regulation via its ETGE and DLG motifs (69, 70). Neh4/5 recruits CBP to promote transcription—a process suppressed by the Neh7–RARα complex (71, 72). The serine-rich Neh6 domain governs ubiquitin-mediated degradation, further regulating Nrf2 stability (73).

Under basal conditions, Keap1 maintains Nrf2 at low levels through constitutive binding. Oxidative stress triggers conformational changes in Keap1 via cysteine residue modifications, disrupting Keap1-Nrf2 interaction (74). Electrophilic inducers similarly modify Keap1 cysteines to promote Nrf2 release (75, 76). Liberated Nrf2 translocates to the nucleus, heterodimerizes with small Maf proteins (MafF, MafG, or MafK in vertebrates), and binds ARE sequences to transactivate cytoprotective genes including heme oxygenase-1 (HO-1) and NAD(P)H quinone oxidoreductase 1 (NQO1) (77, 78). Downstream genes regulated by the Nrf2/ARE axis include HO-1, NQO1, and iron metabolism-related proteins (FTH1, FTL, FPN1), etc.: HO-1 degrades pro-oxidative heme to produce bilirubin with iron-chelating activity, and NQO1 directly scavenges lipid free radicals; both jointly block the lipid peroxidation cascade (79). Iron metabolism proteins reduce intracellular iron accumulation to decrease ferroptosis susceptibility (80, 81). In addition, the autophagic adaptor p62 can bind to Keap1 to promote its autophagic degradation, and at the same time, it is transcriptionally upregulated by Nrf2-ARE signaling, forming a positive feedback regulatory loop to enhance axis function (82, 83).

In the AD pathological microenvironment, the function of the Nrf2/ARE axis is significantly abnormal: the upregulated expression of Keap1 in the brains of AD patients leads to increased Nrf2 degradation. Meanwhile, Aβ and hyperphosphorylated tau can directly inhibit the nuclear translocation and transcriptional activity of Nrf2, impairing the axis’s function in regulating antioxidant and iron metabolic balance. This functional defect exacerbates oxidative stress and neuronal ferroptosis in the AD brain, further promoting Aβ deposition and tau pathology. Conversely, activating Nrf2 can significantly reduce pathological damage in the brains of AD model animals and improve learning and memory abilities by enhancing antioxidant defense and inhibiting ferroptosis. Preclinical studies have shown that Nrf2 activators can exert neuroprotective effects in AD by restoring the function of this axis, highlighting its potential as a therapeutic target for AD.

#### 3.3.3 FSP1-NADH-CoQ axis

Ferroptosis suppressor protein 1 (FSP1), alternatively termed AMID or PRG3, exhibits pleiotropic biological functions. Initially characterized as a mitochondrially localized pro-apoptotic factor, *in vitro* and *in vivo* experiment reveal that myristoylation-mediated translocation to the plasma membrane converts FSP1 into a potent ferroptosis inhibitor (84, 85). The core molecular mechanism involves NAD(P)H-dependent reduction of coenzyme Q10 (CoQ) to ubiquinol (CoQH_2_), which directly quenches lipid radicals through its antioxidant activity (84). Beyond its classical CoQ reductase function, FSP1 additionally suppresses ferroptosis via repair of oxidatively damaged plasma membranes and activation of the noncanonical vitamin K cycle (86). These findings not only elucidate the sophisticated defense network orchestrated by the FSP1-NAD(P)H-CoQ axis but also provide novel conceptual frameworks for developing ferroptosis-targeted interventions.

FSP1 expression is directly transcriptionally regulated by Nrf2, while Nrf2’s downstream target NAD(P)H quinone dehydrogenase 1 (NQO1) cooperatively maintains the reduced state of CoQ10 (87). Conversely, the antioxidative effects generated by the FSP1-CoQ10 system mitigate oxidative stress, indirectly stabilizing Nrf2 protein levels (84). This reciprocal positive feedback regulation establishes a sustained cellular defense system via the Nrf2-FSP1-CoQ10 axis, providing critical compensatory protection particularly during GPX4 inactivation. Significantly, this pathway functionally synergizes with the vitamin E antioxidant system through complementary membrane lipid protection mechanisms, offering a rational basis for combination therapeutic strategies against ferroptosis-associated pathologies.

### 3.4 Mitochondria in ferroptosis

Mitochondria, central to eukaryotic cellular iron metabolism, maintain iron homeostasis primarily through heme and ferritin storage. Relevant cell experiments reveal that this unique iron sequestration capacity positions mitochondria as contributory organelles in regulating ferroptosis (88). In Friedreich’s ataxia (FRDA) models, mitochondrion-specific iron overload directly triggers ferroptosis, while the mitochondrially targeted antioxidant XJB-5–131 significantly mitigates this process through chelation of labile iron (89). Further investigations demonstrate that mitochondrial-specific iron-sulfur (Fe-S) cluster biogenesis also modulates ferroptosis susceptibility. These findings collectively establish mitochondria as indispensable regulators within the ferroptosis network (90, 91).

Mitochondrial fatty acid oxidation generates acetyl-CoA, a major substrate for the TCA cycle. This process yields NADH and FADH_2_, which provide electrons to the electron transport chain (ETC). Oxidative phosphorylation (OXPHOS) depends on iron ions as essential cofactors in ETC complexes, including cytochrome c oxidase, where they participate directly in electron transfer and oxygen reduction. Concurrently, elevated mitochondrial iron promotes peroxidative damage through Fenton-type reactions, ultimately triggering ferroptosis (88, 92). Moreover, mitochondrial iron overload inhibits Fe-S cluster-dependent enzymes such as aconitase 2, leading to the accumulation of metabolites like succinate. Excess succinate drives reverse electron transfer at complex I, while impaired α-ketoglutarate dehydrogenase activity limits NADPH regeneration. Together, these alterations weaken cellular antioxidant capacity (93). The resulting interplay between metabolic dysregulation and oxidative stress establishes a self-amplifying cycle, rendering ferroptosis largely irreversible once initiated.

Significantly, cardiolipin (CL), enriched in mitochondrial inner membranes with exceptionally high PUFA content (>80%), serves as a preferred target for peroxidation during ferroptosis (94). Under ROS attack, CL readily undergoes non-enzymatic peroxidation, leading to molecular degradation, double-bond reduction, increased hydrophilicity, and altered negative charge density. These modifications impair essential biological processes including electron transport, TCA cycling, and iron metabolism (95). Significantly, cardiolipin peroxidation products (e.g., hydroxyalkenal-modified CL) act as specific signaling molecules promoting cytochrome c (Cyt c) dissociation from the inner membrane and subsequent cytosolic release, constituting a event in mitochondrial apoptosis initiation.

In summary, dysregulated mitochondrial iron metabolism, ETC dysfunction, and cardiolipin peroxidation collectively regulate ferroptosis execution. Targeting mitochondrion-specific pathways—such as ETC inhibition, cardiolipin protection, or iron chelation—demonstrates anti-ferroptotic efficacy in neurodegenerative disease models, highlighting their therapeutic potential. These advances substantially advance our mechanistic understanding of ferroptosis while offering novel perspectives for targeted therapeutic approaches in neurodegeneration. Future research should prioritize elucidating mitochondrial coordination with other organelles (e.g., lysosomes) in ferroptosis and investigating how organelle-specific mitochondrial signatures influence ferroptosis susceptibility.

## 4 Ferroptosis and other forms of cell death

Ferroptosis is an iron-dependent form of cellular necrosis driven by the abnormal accumulation of lipid peroxides. Its core lies in the failure of the cellular antioxidant defense system, leading to the buildup of lipid ROS and ultimately disrupting plasma membrane integrity. To more clearly define this unique form of cell death and understand its complex roles in physiological and pathological processes, the following will systematically distinguish ferroptosis from three other important cellular events—apoptosis, necroptosis, and autophagy—in terms of their fundamental differences in core mechanisms, morphological features, and functional impacts.

### 4.1 Ferroptosis and apoptosis

Both are forms of regulated cell death, but they differ significantly in their execution mechanisms and morphological features. Apoptosis is a classic form of programmed cell death that relies on the cascade activation of caspase proteases. It plays an important role in maintaining tissue homeostasis and coordinating immune and defense functions (96, 97). Morphologically, apoptosis is characterized by cell shrinkage, chromatin condensation, and the formation of apoptotic bodies. Notably, the entire process occurs without the leakage of cellular contents and does not induce an inflammatory response. In contrast, ferroptosis does not depend on caspase activation. Its core driving factor is iron-mediated lipid peroxidation. Morphologically, ferroptosis mainly manifests as disruption of plasma membrane integrity, cytoplasmic vacuolization, and specific mitochondrial alterations (such as volume reduction, increased membrane density, and diminished or absent cristae), without the formation of apoptotic bodies. The release of cellular contents can trigger a local inflammatory response.

Under specific conditions, cells can switch from apoptosis to ferroptosis, a transition that enhances cellular sensitivity to apoptotic signals (98, 99). For example, when cancer cells develop resistance to apoptosis (e.g., due to high expression of BCL-2 protein or Caspase inactivation), inducing ferroptosis can serve as an effective alternative strategy to eliminate these cells. Additionally, some upstream stress signals (such as p53 activation) can trigger apoptosis and, under specific conditions (e.g., by inhibiting SLC7A11), promote ferroptosis (100, 101). Therefore, the intersection of ferroptosis and apoptosis pathways provides new insights for cancer therapy.

### 4.2 Ferroptosis and necroptosis

The distinction between ferroptosis and necroptosis lies mainly in their dependency mechanisms and triggering factors. Although both are forms of necrotic cell death that lead to plasma membrane rupture and inflammatory responses, their core mechanisms differ. Necroptosis is a programmed necrosis triggered by cytokines such as tumor necrosis factor (TNF) and relies on receptor-interacting protein kinase 1 (RIPK1), RIPK3, and mixed lineage kinase domain-like protein (MLKL). Its key step involves kinase cascade-mediated activation and oligomerization of MLKL, which subsequently disrupts plasma membrane integrity (102). In contrast, ferroptosis is not associated with cytokine signaling. Its core depends on the presence of iron ions and the accumulation of lipid peroxidation and does not involve the RIPK1/RIPK3/MLKL pathway. Furthermore, necroptosis can be inhibited by specific agents such as necrostatin-1, whereas ferroptosis inhibition primarily relies on iron chelators (e.g., deferoxamine) or GPX4 activators, making their inhibitors entirely different.

Studies have shown that a key link between ferroptosis and necroptosis pathways is their sharing of some upstream stress sensors (103). For example, certain kinases or oxidative stress signals can simultaneously activate or regulate both pathways. Müller et al. further proposed that ferroptosis and programmed necrosis are complementary mechanisms of cell death (104). It is known that the key lipid metabolism enzyme ACSL4 serves as an indicator of ferroptosis, while MLKL, involved in the execution of necrosis, is a marker of programmed necrosis (102, 105). A deficiency in ACSL4 can lead to upregulation of MLKL expression, making cells more sensitive to ferroptosis (106). This evidence collectively suggests a dynamic compensatory relationship between the two pathways: inhibiting one cell death mechanism may lead to the activation of the other as compensation. Therefore, in therapeutic strategies, combined inhibition of both pathways may provide stronger tissue protection than inhibiting either one alone.

### 4.3 Ferroptosis and autophagy

Autophagy itself is a cellular self-protection mechanism. Its core is the autophagy-related gene (ATG)-regulated lysosomal degradation pathway, which degrades and recycles damaged organelles or proteins by forming autophagolysosomes to cope with stress and maintain homeostasis (107, 108). Under physiological conditions, autophagy plays a protective role in maintaining cellular homeostasis and may induce cell death only when it is excessively activated or when the autophagic flux is blocked. Under specific conditions, excessive or dysregulated autophagy can lead to cell death, but this is generally considered an “abnormal” consequence of autophagy.

*In Vitro* studies have revealed a new role for autophagy in cancer cell death, indicating that autophagy can trigger ferroptosis by initiating ferritin decomposition (109, 110). Specifically, autophagy can promote ferroptosis through selective degradation of ferritin (ferritinophagy) or antioxidant proteins. However, in most cases, autophagy remains a cell survival mechanism. Therefore, autophagy is not a specific death execution program; it primarily functions as an upstream regulatory factor to modulate ferroptosis, whereas ferroptosis is a well-defined death execution pathway culminating in lipid peroxidation. These findings reveal the intricate relationship and mutual regulatory mechanisms between ferroptosis and autophagy, providing new insights into understanding the complex network of cell death pathways and their interdependencies (110).

## 5 Ferroptosis and the core pathology of AD

### 5.1 Ferroptosis and Aβ plaques

Aβ misfolding and aggregation are key to AD pathology, with iron dysregulation promoting ferroptosis (111). Early AD involves iron accumulation in cortical and hippocampal areas (112), which disrupts iron homeostasis by altering key regulators: TfR1 and DMT1 decrease, while ferritin, ferroportin 1, and APP increase (113, 114). This enhances iron retention and shifts APP processing toward Aβ production (115). The aberrantly processed APP forfeits its chaperone-like function, further impairing ferroportin 1-mediated iron export and establishing a self-perpetuating cycle of iron dyshomeostasis (10, 116). Crucially, Soluble Aβ also binds Fe³^+^, reducing it to Fe²^+^ and accelerating plaque formation via iron-mediated oxidation (117). Together, these processes link iron dyshomeostasis, Aβ toxicity, and ferroptosis in driving neurodegeneration.

A bidirectional regulatory network exists between Aβ deposition and ferroptosis. Significant accumulation of lipid peroxides is observed in brain regions enriched with Aβ oligomers and in 4-HNE-positive areas of AD patients, indicating direct involvement of Aβ in the ferroptosis pathway (118). A core pathogenic mechanism involves Aβ oligomers embedding into the lipid bilayer of cell membranes, disrupting the spatial conformation of phospholipid dehydrogenases. This conformational impairment reduces dehydrogenation efficiency, non-enzymatically accelerating free radical chain reactions and lipid peroxidation (119). Pathological Aβ can also increase neuronal susceptibility to ferroptosis by activating the oxytocin receptor signaling axis (OXTR/PI3K/AKT pathway) (120). Conversely, the ferroptosis pathway exerts feedback on Aβ pathology. Activation of the upstream regulator peroxisome proliferator-activated receptor α (PPARα) induces the expression of ferroptosis suppressor protein 1 while simultaneously downregulating APP synthesis and inhibiting β-site APP cleaving enzyme 1 hydrolytic activity, effectively reducing Aβ oligomer generation (121). Furthermore, coenzyme Q10, an essential electron carrier in the FSP1 pathway, directly blocks lipid peroxidation chain reactions through its endogenous antioxidant properties and mitigates Aβ pathological burden in the cortex and hippocampus (122). Taken together, these findings illustrate a plausible and complex bidirectional interplay between Aβ pathology and ferroptosis-associated lipid peroxidation. However, a critical distinction must be made between mechanistic associations established in experimental systems and causative evidence in human disease. To date, conclusive evidence substantiating ferroptosis as a primary pathogenic driver in human AD is still lacking. Future research employing longitudinal biomarker studies and targeted interventional trials in human populations will be essential to validate its causal role and therapeutic relevance.

### 5.2 Ferroptosis and tau protein

Imbalanced brain iron homeostasis is mechanistically linked to tau hyperphosphorylation—a hallmark of AD pathology. Under physiological conditions, tau stabilizes microtubules and supports axonal transport. In AD, however, tau undergoes pathological hyperphosphorylation, leading to its dissociation from microtubules, aggregation into neurofibrillary tangles, and ultimately neuronal dysfunction (1).

Iron overload is hypothesized to exacerbate this process through several pathways. Experimentally, excess iron can impair insulin signaling, inhibit PI3K/Akt activity, and thereby enhance glycogen synthase kinase 3β-mediated tau phosphorylation at residues including Ser202, Thr205, and Ser396/404 (123). Iron may also promote cyclin-dependent kinase 5 activation via p25 binding, further driving tau phosphorylation (124). Additionally, iron-activated ERK/MAPK pathways may contribute to tau phosphorylation and neuroinflammatory cytokine release, suggesting a potential feedback loop between iron dysregulation, tau pathology, and inflammation (125, 126). Notably, reduced soluble tau levels have been associated with decreased expression of the iron exporter ferroportin 1, potentially worsening cerebral iron retention (127).

Phosphorylated tau may also intersect with lipid peroxidation processes relevant to ferroptosis. In AD models, phospho-tau colocalizes with lipid rafts enriched in polyunsaturated fatty acids, and ω-6 fatty acids such as arachidonic acid have been shown to promote tau oligomerization (128, 129). More critically, phosphorylated tau can electrostatically interact with anionic phospholipids, potentially destabilizing membranes and amplifying lipid peroxidation via NADPH oxidase activation (e.g., NOX2), thereby linking tau pathology to ferroptotic membrane damage (130).

While these experimental observations outline a plausible network connecting iron dysregulation, tau pathology, and ferroptosis, it is essential to distinguish between mechanistic associations established in model systems and causative evidence in human disease. Current support for ferroptosis as a primary driver of AD pathogenesis remains largely correlative, relying on post-mortem analyses, animal models, and *in vitro* data. Conclusive evidence from longitudinal clinical studies or targeted interventional trials in humans is still lacking. Thus, the role of ferroptosis in human AD—particularly as an initiating pathogenic event—requires further validation through clinically integrated research approaches.

### 5.3 Ferroptosis and neuroinflammatory plaques

#### 5.3.1 Microglial cells

Microglia, the resident immune cells of the central nervous system, constitute approximately 10–12% of total brain cells and play a significant role in maintaining immune surveillance and neural homeostasis (131, 132). Beyond their functions in pathogen clearance and metabolic waste removal, microglia actively support neurodevelopment, synaptic plasticity, and neuroprotection (133, 134). It is important to note, however, that while most *in vivo* studies support a neuroprotective role for activated microglia, evidence from ex vivo or cultured microglial models suggests these cells may also exhibit neurotoxic potential. This phenotypic transition—from a protective, surveillant state to a pro-inflammatory and potentially detrimental phenotype—represents a key mechanism underlying neuroinflammation and neurodegeneration in AD.

##### 5.3.1.1 Microglial and ferroptosis

Elevated cerebral iron levels activate the NF-κB signaling pathway, initiating the classical pro-inflammatory phenotypic transformation of microglia (135). Concurrently, iron acts as a damage-associated molecular pattern that can be recognized by microglial Toll-like receptor family members (e.g., TLR4), further amplifying inflammatory signaling. In response to the extracellular high-iron milieu, activated microglia upregulate ferritin expression in an attempt to sequester iron. Paradoxically, this promotes intracellular iron retention and oxidative stress, exacerbating the inflammatory microenvironment (136). Ferritin is a cage-like protein complex composed of 24 H- and L-subunits, whose stoichiometry is dynamically regulated in microglia to meet functional demands. Studies show that in high-iron conditions, microglia specifically upregulate L-subunit expression, and ferritin expression is observed predominantly under pathological conditions of significant brain iron elevation (137). This upregulation of microglial ferritin is a direct response to excess free iron released during neuronal ferroptosis. When early tangle-bearing neurons undergo ferroptosis, the released free iron triggers neighboring microglia to initiate ferritin synthesis, aiming to lower extracellular iron concentration through intracellular sequestration (138, 139). Consequently, the spatial distribution of immunoreactive ferritin-positive microglia can serve as a biological marker for the degree of labile iron accumulation in the brain parenchyma (139).

Neurons and microglia differ in iron handling: neurons rely on iron for metabolic activity, while microglia normally use ferritin storage for neuroprotection. In AD, however, elevated brain iron drives microglia toward a ferroptosis-prone state, marked by upregulated heme oxygenase-1 and enhanced release of inflammatory mediators like IL-1β (140, 141). This shift further accelerates Aβ accumulation via NADPH oxidase activity (142). The resulting oxidative environment induces lipid peroxidation and DNA damage, while activating MAPK/NF-κB signaling to promote amyloid precursor protein expression and impair Aβ clearance. Heme breakdown by HO-1 releases free iron, directly fueling iron-dependent peroxidation (140, 141). Together, iron and oxidative mediators activate the NLRP3 inflammasome, increasing IL-1β release and establishing a self-amplifying loop linking iron overload, neuroinflammation, and ferroptosis (18).

Activated immune pathways can positively regulate the susceptibility of microglia to ferroptosis and amplify their neurotoxicity. For instance, the activation of NF-κB and NLRP3 pathways leads to massive cytokine release (143). These cytokines, by inhibiting neuronal system Xc^-^ function and activating ACSL4, synergistically drive neuronal membrane lipid peroxidation and directly induce neuronal ferroptosis. Activated microglia can also generate potent oxidants like peroxynitrite, which inhibit key anti-ferroptotic proteins such as GPX4 and SLC7A11, dismantling the antioxidant defense systems of both the microglia themselves and neighboring neurons. Ultimately, ferroptotic microglia release damage-associated molecular patterns (DAMPs), including free iron, oxidized phospholipids, and HMGB1 (140, 144). These DAMPs can be sensed by surrounding viable microglia via pattern recognition receptors like TLRs, thereby re-initiating a new cycle of immune activation and inflammatory response.

##### 5.3.1.2 Microglia and AD

The pathological significance of activated microglia in the brains of AD patients was first systematically described by McGeer and colleagues in 1987, a discovery that ignited extensive investigation into their potential role in neurodegenerative progression. The traditional view posits that microglia activated around senile plaques accelerate disease progression by releasing ROS, neurotoxins, and pro-inflammatory cytokines, thereby directly linking Aβ deposition to neurodegeneration (145, 146). Studies confirm that ferritin-positive microglia can emerge during the preclinical stage of LOAD. Their numbers increase with age and they form clusters around blood vessels and within senile plaque regions (147, 148). The density of these cells positively correlates with the severity of tau pathology, and their clustered aggregation is often a harbinger of neuritic plaque formation (149, 150). Consequently, microglia are regarded as key immune effector cells that drive the maturation of Aβ plaques and exacerbate the development of neurofibrillary pathology (151).

##### 5.3.1.3 Microglial atrophy and AD

The recently proposed concept of “microglial atrophy” describes characteristic structural alterations in these cells during aging, including somatic shrinkage and process reduction (152). This phenotype is closely associated with upregulated intracellular ferritin expression, elevated brain iron levels, and oxidative stress. Data from AD patients and aged animal models demonstrate a significant decline in Aβ clearance capacity in senescent microglia (153, 154). This indicates that impaired microglial phagocytic function is a significant component of AD pathogenesis and suggests that restoring their homeostatic function through targeted intervention could be a potential therapeutic strategy (152).

In late-stage AD brain tissue, most microglia appear in a quiescent or dystrophic state, lacking classic activation markers such as soma hypertrophy. Similarly, microglia surrounding early neuronal ferroptosis events triggered by tau pathology frequently show no typical activation or phagocytic response. This functional deficit contrasts sharply with the normal physiological role of microglia, which is to rapidly respond to neuronal injury signals and clear cellular debris. This functional loss may stem from age-related phagocytic exhaustion and the direct toxicity of iron-rich Aβ and tau aggregates toward microglia (155) Furthermore, prior cell culture studies confirm that amyloid exposure accelerates microglial senescence (156). Based on this, we can hypothesize that iron-mediated oxidative toxicity may specifically damage microglia, thereby inhibiting their capacity to clear pathological proteins.

Despite showing atrophic damage, ferritin-positive microglia can maintain basic functions, a resilience attributed to their unique oxidative stress tolerance (157). These cells possess a hierarchical antioxidant system comprising superoxide dismutase, catalase, and glutathione peroxidase, and they maintain higher basal glutathione levels than neurons and astrocytes (157). This intrinsic antioxidant advantage enables microglia to preserve functional integrity under oxidative pressure (158, 159). Further research shows that lipopolysaccharide and interferon-γ stimulation can reduce glutathione levels in microglia, which inversely confirms the critical role of high glutathione reserves in their antioxidant capacity under resting conditions (160). Therefore, in the oxidative stress environment of AD, microglia can achieve self-preservation and functional maintenance by dynamically upregulating their antioxidant defenses.

While experimental understanding continues to advance, the precise causal relationship between microglial activation, neuroinflammation, and Alzheimer’s disease progression in humans remains to be fully established. It is noteworthy that although dysregulated microglial function is closely associated with AD pathology—including interactions with Aβ, tau, and emerging processes such as ferroptosis—the hypothesis that microglia-driven neuroinflammation or related ferroptosis serves as an initiating or central driver of human AD still relies predominantly on correlative and preclinical evidence. Further longitudinal clinical studies and targeted interventional trials are necessary to determine whether modulating microglial activity or ferroptosis can meaningfully alter the disease course in AD patients.

#### 5.3.2 Astrocytes

The entry of dietary iron into the central nervous system is contingent upon blood-brain barrier(BBB) integrity, which is frequently compromised in Alzheimer’s disease. Large-scale postmortem analyses indicate that cerebrovascular pathology occurs in approximately 80% of AD cases—a prevalence markedly higher than in other neurodegenerative disorders such as Parkinson’s disease, frontotemporal dementia, and Lewy body disease—highlighting a potentially distinctive role of vascular dysfunction in AD pathogenesis (161).

Astrocytes, the predominant glial cell type in the brain, are strategically positioned at the BBB interface and contribute critically to cerebral homeostasis, neuronal support, and immune modulation (162, 163). In AD, astrocytes undergo activation and are implicated in both initiating and sustaining neuroinflammatory responses. They participate in the fine-tuned regulation of iron uptake, storage, and release—a process partly mediated by ceruloplasmin—to balance neuronal iron availability while limiting its toxicity (164–166). However, the molecular pathways governing astrocytic iron transport, including the contextual regulation of DMT1, ferroportin 1, and transferrin receptor 1, remain incompletely resolved and may vary with pathological conditions (167, 168). Under iron-rich conditions, astrocytes can enhance antioxidant defenses, such as glutathione synthesis, to maintain cellular viability (169). Yet, during prolonged inflammatory stimulation, they may adopt a pro-inflammatory phenotype, releasing substantial quantities of cytokines and thereby amplifying neuroinflammatory cascades (170, 171).

Although astrocyte dysfunction and iron dysregulation are increasingly recognized in AD pathology, their direct causal link to disease progression—and particularly to hypothesized central mechanisms such as ferroptosis—requires further validation in human contexts. Current evidence remains largely correlative or derived from preclinical models. Thus, while astrocytes appear to occupy a key intersection between iron metabolism and neuroinflammation in AD, conclusive evidence establishing ferroptosis as a primary pathogenic driver in human disease is still lacking. Future studies integrating longitudinal clinical data and mechanistic interventional approaches will be essential to clarify these relationships and their therapeutic relevance.

#### 5.3.3 Brain capillary endothelial cells

Brain capillary endothelial cells (BCECs), a central component of the blood-brain barrier, regulate cerebral iron uptake through distinct yet possibly complementary transport models. Two principal mechanisms have been proposed: the classical transferrin-dependent pathway and the transcytosis model (39). The classical pathway describes a sequence in which transferrin-bound iron enters BCECs via receptor-mediated endocytosis. Within acidic endosomal compartments, Fe³^+^ is reduced to Fe²^+^, exported into the cytosol by divalent metal transporter 1 (DMT1), and subsequently released across the abluminal membrane via ferroportin 1 (FPN1), followed by re-oxidation through ceruloplasmin before entering the brain parenchyma (172, 173).

In contrast, the transcytosis model posits that the transferrin-iron complex undergoes vesicular trafficking across the endothelial cytoplasm without cytosolic release through DMT1 (174). This model is supported by observations of minimal DMT1 expression in BCECs, suggesting that iron may transit directly within endosomal vesicles to reach the brain interstitium (175, 176). These two models are not mutually exclusive and may operate under varying physiological or pathological conditions, though their interplay remains to be fully elucidated.

It should be noted that current understanding of iron transport mechanisms in brain capillary endothelial cells is predominantly derived from *in vitro* models or specific animal studies, and their applicability to intact biological systems and human physiological environments remains to be fully validated. Particularly noteworthy is the lack of direct human tissue evidence and dynamic functional studies regarding how these transport pathways are altered and contribute to pathology in neurodegenerative diseases such as Alzheimer’s disease. Furthermore, existing research has largely focused on transport mechanisms under physiological conditions, while in pathological contexts—such as impaired blood-brain barrier integrity or chronic inflammatory microenvironments—these transport patterns may undergo significant reprogramming, with regulatory networks likely far more complex than current models suggest.

## 6 Evidence of ferroptosis in AD

### 6.1 Imaging evidence

Recent advancements in neuroimaging have significantly advanced the diagnosis and research of AD. Multimodal imaging has become a cornerstone, with clinical guidelines recommending structural neuroimaging in cognitively impaired individuals to assess medial temporal lobe atrophy and rule out other pathologies (177, 178). Post-mortem studies provide the most direct evidence, demonstrating significant iron accumulation within glial cells surrounding Aβ plaques in the AD brain, suggesting iron overload as a key driver of plaque formation. For *in vivo* imaging, quantitative susceptibility mapping (QSM), an advanced MRI technique, enables precise quantification of regional brain iron deposition. Studies show that QSM-derived iron levels correlate significantly and negatively with cognitive function in AD patients, confirming the role of iron dysregulation in the disease mechanism (11, 179). Integrated analyses combining amyloid-PET with QSM further indicate that elevated cerebral iron load is closely associated with Aβ-related cognitive decline, suggesting a synergistic neurotoxic interaction between the two pathological processes.

Beyond conventional structural MRI and molecular imaging, emerging technologies such as diffusion tensor imaging (DTI), arterial spin labeling (ASL), and novel PET tracers targeting the cholinergic system or microglial activation provide unprecedented insights into AD pathophysiology (180). Although automated image analysis techniques require further refinement, these imaging advances not only deepen our understanding of ferroptosis-related mechanisms in AD but also lay the groundwork for developing early diagnostic and targeted therapeutic strategies (181, 182). Notably, QSM-based iron quantification holds strong potential to become a key component of future multi-modal AD biomarker panels.

### 6.2 Pathological evidence

**Figure 3:**
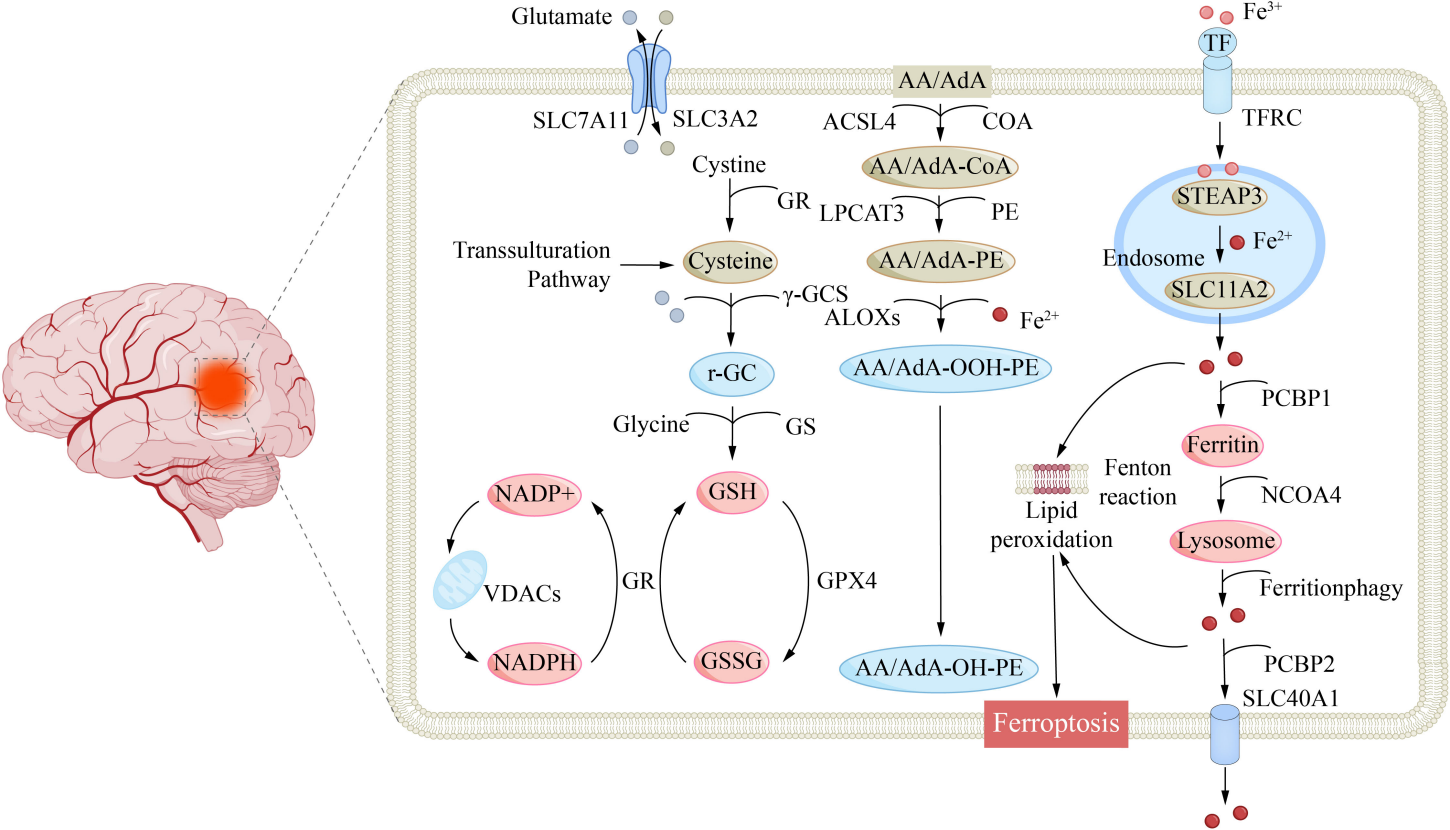
Molecular mechanisms of ferroptosis in brain microenvironment. Extracellular glutamate accumulates and inhibits the cystine-glutamate antiporter SLC7A11/SLC3A2, blocking cystine uptake and starving the cell of cysteine needed for glutathione (GSH) synthesis; simultaneously, transferrin receptor (TFRC) imports Fe³^+^ into endosomes where STEAP3 reduces it to Fe²^+^ that is released into the cytosol via DMT1/SLC11A2, and cytosolic chaperones PCBP1/2 deliver Fe²^+^ to ferritin or mitochondrial VDACs; NCOA4-mediated ferritinophagy degrades ferritin, liberating redox-active Fe²^+^ that fuels Fenton chemistry to generate hydroxyl radicals, which together with ALOX enzymes convert polyunsaturated fatty-acid phospholipids (AA/AdA-PE) to lipid hydroperoxides (AA/AdA-OOH-PE); because the GPX4-GSH axis is incapacitated by GSH depletion, these lipid peroxides cannot be reduced, accumulate, and ultimately rupture membranes, executing ferroptosis.

Goodman first histochemically identified abnormal iron deposition in the cerebral cortex of AD patients in 1953 (183). Subsequent studies confirmed elevated iron levels in AD gray matter (184), with regional accumulation particularly evident in cognitive-related limbic and paralimbic structures such as the amygdala, hippocampus, and olfactory pathways (185). Recent evidence indicates that AD brains exhibit canonical ferroptotic pathology, which drives mitochondrial cardiolipin peroxidation, causes ultrastructural damage, impairs ATP synthesis, and ultimately triggers irreversible neurodegenerative decline (**Figure 3**) (186).

#### 6.2.1 GSH/GPX4

Depleted glutathione levels in the hippocampus and frontal cortex are strongly associated with severe cognitive impairment in Alzheimer’s disease (18). In AD rat models, N-acetylcysteine exerts anti-lipid peroxidation effects by elevating brain GSH levels, with its neuroprotective mechanism linked to enhanced cellular and mitochondrial membrane stability (187, 188). Post-mortem brain tissue from AD patients and transgenic mouse models (e.g., APP/PS1, 5xFAD) consistently shows significant downregulation of glutathione peroxidase 4 protein expression, accompanied by characteristic ferroptotic morphological alterations such as mitochondrial shrinkage (189). As a key enzyme responsible for clearing peroxides, GPX4 inactivation can directly lead to hippocampal neuronal death. Experimentally, mice with conditional knockout of GPX4 in the cortex and hippocampus exhibit profound spatial memory deficits and hippocampal neurodegeneration (189).

**Figure 4:**
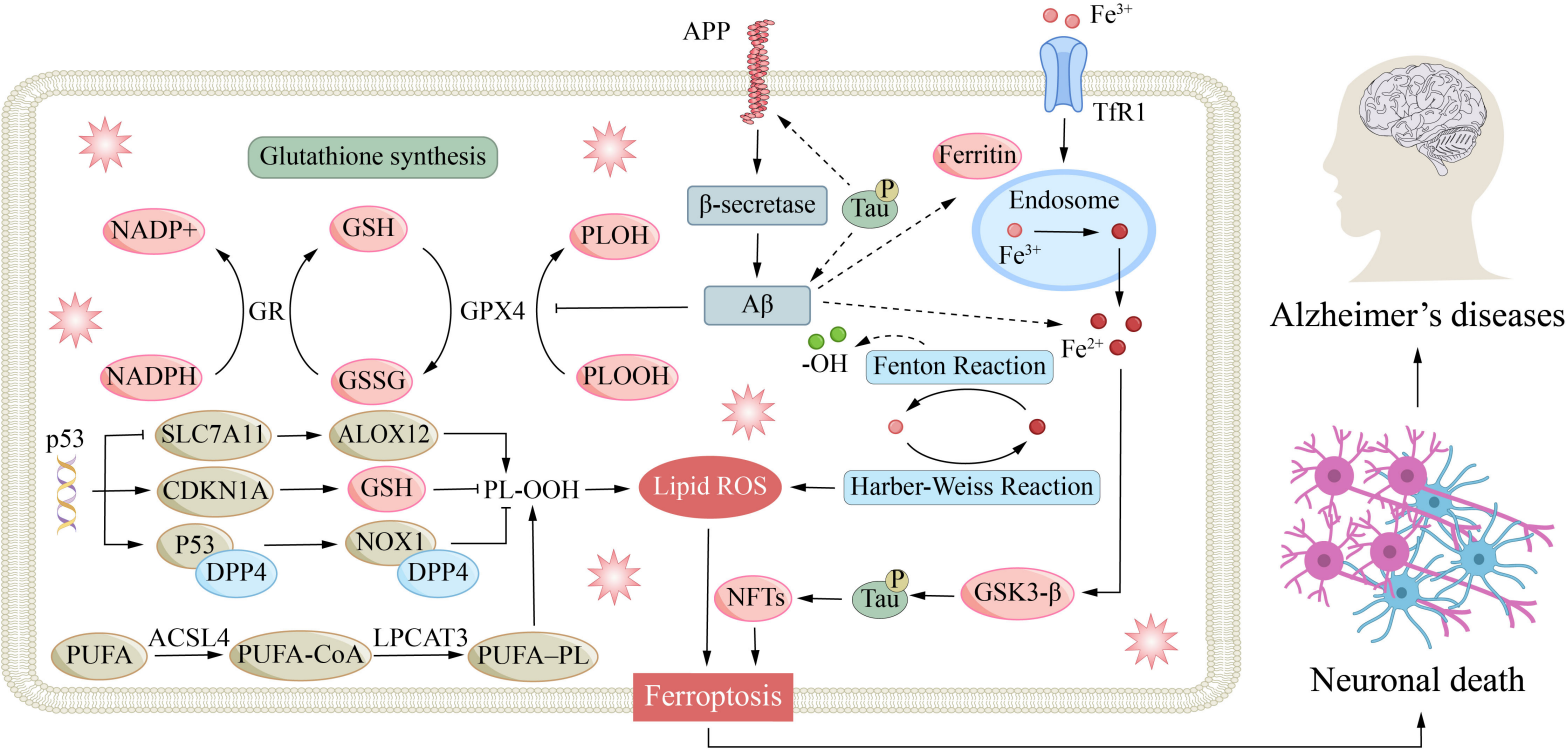
Mechanisms associated with ferroptosis in AD. APP is cleaved by β-secretase to produce Aβ oligomers that trigger hyperphosphorylation of Tau into neurofibrillary tangles (NFTs) and activate p53, which up-regulates CDKN1A and DPP4, represses SLC7A11, and thereby lowers GSH synthesis and inactivates GPX4; Aβ also promotes TfR1-mediated endocytosis of Fe³^+^ that is reduced to Fe²^+^ in endosomes and released via DMT1, and the resulting labile iron generates hydroxyl radicals through Fenton and Haber-Weiss reactions that enable ALOX12 to convert PUFA-containing phospholipids (PUFA-PL) to lipid hydroperoxides (PL-OOH); with the GSH-GPX4 antioxidant axis collapsed and lipid ROS relentlessly accumulating, neuronal membranes undergo oxidative demolition that triggers ferroptosis and accelerates Alzheimer’s disease progression.

Mechanistic studies reveal that lipoic acid can inhibit ferroptosis by downregulating transferrin receptor expression and upregulating GPX4, thereby protecting neurons involved in learning and memory and improving cognitive function (**Figure 4**) (190). This regulatory relationship is inversely validated at the molecular level: the GPX4 inhibitor RSL3 potently induces ferroptosis by binding to its active site, serving as a reliable experimental tool for studying ferroptosis in AD and directly establishing a causal link between GPX4 inhibition and AD pathology (191). Targeting the GPX4 pathway has proven effective in mitigating AD-associated ferroptosis. In both *in vitro* and *in vivo* models, ferroptosis inhibitors significantly reduce Aβ-aggregation-induced neuronal death and ameliorate memory deficits (44). Similarly, PPARα agonists alleviate lipid peroxidation and neuroinflammation by enhancing GPX4 transcription (192). These collective findings confirm that GPX4 dysfunction exacerbates AD progression by promoting ferroptosis. This not only deepens our mechanistic understanding of the disease but also highlights the significant therapeutic potential of modulating the GSH-GPX4 axis in AD intervention.

#### 6.2.2 Biomarkers of lipid peroxidation

Lipid peroxidation, a core pathological hallmark of ferroptosis, manifests prominently in AD. Studies using the 5xFAD transgenic mouse model demonstrate significantly elevated levels of lipid peroxidation end products (MDA, 4-HNE) and secondary products like lysophospholipids in brain tissue (189). This phenomenon is further validated in clinical samples, where MDA and 4-HNE levels are markedly increased in the brain tissue, cerebrospinal fluid, and blood of AD patients compared to healthy controls, with post-mortem analyses providing additional confirmation (193). Notably, 4-HNE can activate the PPARγ pathway, suppressing PGC-1α-mediated mitochondrial biogenesis and reducing local ATP supply at synapses, thereby impairing plasticity mechanisms such as LTP (56) Simultaneously, the electrophilic properties of 4-HNE facilitate its formation of adducts with synaptic proteins (e.g., synapsin I, PSD-95), directly disrupting synaptic ultrastructure (194). Under this dual assault of energy metabolism impairment and membrane structural damage, synapses emerge as vulnerable early targets of ferroptosis, providing a mechanistic basis for the cognitive alterations observed in the preclinical stages of AD.

Critically, key cellular metabolic pathways—including thiol metabolism, fatty acid metabolism, and mitochondrial respiration—directly influence cellular susceptibility to lipid peroxidation and ferroptosis (195). In transgenic AD mouse models, the fatty acid synthase inhibitor CMS121 prevents excessive lipid peroxidation and inflammation while mitigating cognitive decline (196). Mitochondrial aldehyde dehydrogenase (ALDH2) ameliorates dual cardiac and neurological dysfunction in APP/PS1 mice by degrading toxic aldehydes and inhibiting ACSL4-mediated ferroptosis (197).

### 6.3 Classical genetic risk factors for AD

#### 6.3.1 APOEϵ4

APOE represents one of the most prominent genetic risk factors for AD, with its ϵ4 allele (APOEϵ4) demonstrating a strong association with disease pathogenesis and progression. In mouse models has established that APOE suppresses HMG-CoA reductase within the cholesterol synthesis pathway, leading to substantial intracellular accumulation of acetyl-CoA, the precursor for cholesterol biosynthesis (198). Given that acetyl-CoA also serves as the precursor for PUFA synthesis, its excessive accumulation provides the substrate foundation for lipid peroxidation, thereby fostering a pro-ferroptotic microenvironment. Clinical cohort studies confirm that cerebrospinal fluid levels of lipid peroxidation markers (e.g., MDA, 8-OHdG) are significantly elevated in APOEϵ4 carriers compared to APOEϵ2 carriers, exhibiting a strong positive correlation with the rate of hippocampal atrophy (199). This underscores the therapeutic potential of targeting the APOE-lipid metabolism axis to disrupt the ferroptotic cascade in AD. Furthermore, APOEϵ4 directly promotes cerebral iron accumulation—positively correlated with cognitive decline—thereby amplifying neurotoxicity and contributing significantly to AD incidence (200, 201).

#### 6.3.2 IREB2

IREB2, an iron-regulatory protein, maintains cellular iron homeostasis by binding to iron-responsive elements and controlling the expression of iron-metabolism genes (202). Loss-of-function mutations in IREB2 impair its inhibition of transferrin receptor 1 expression. Genetic variations in IREB2 have been associated with increased Alzheimer’s disease risk, underscoring its role in AD pathogenesis (203).

#### 6.3.3 PSEN

Mutations in the PSEN1 or PSEN2 genes, associated with familial Alzheimer’s disease, disrupt the maturation of selenocysteine tRNA, leading to impaired cellular selenium uptake. Given that GPX4 is an essential selenoprotein whose activity is wholly dependent on selenium bioavailability, these mutations result in a progressive decline in GPX4 expression. This severely compromises the neuron’s capacity to eliminate lipid peroxides, ultimately driving neurons toward ferroptosis (204).

### 6.4 Epigenetic modifications

Epigenetic modifications serve as an interface between the genome and the external environment. Alterations in external conditions—such as dietary factors, chemical exposures, and psychological stressors—or cellular stress can induce changes in epigenetic marks, leading to gene transcription activation or silencing. These modifications critically regulate fundamental cellular processes including normal growth, development, and the maintenance of gene function. Key epigenetic regulatory mechanisms implicated in the onset and progression of neurodegenerative diseases encompass DNA methylation, histone modifications, and RNA editing (205).

#### 6.4.1 Histone modifications

The activity of β-site APP cleaving enzyme 1 (BACE1), a contributory enzyme for Aβ generation, is transcriptionally regulated by histone acetylation. Specifically, elevated H3 acetylation within the BACE1 promoter enhances its expression, thereby accelerating Aβ deposition (206, 207). Concurrently, aberrant acetylation of tau protein impedes its degradation, promoting neurofibrillary tangle formation and undermining microtubule stability during AD progression. Furthermore, AD brains exhibit dysregulated histone methylation patterns, including alterations in H3K4me3 levels (208). Notably, the methyltransferase G9a catalyzes H3K9 methylation to upregulate key ferroptosis-suppressor genes such as GPX4 and SLC7A11, which may confer neuroprotection in AD (209). n a complementary manner, the demethylase KDM3B inhibits ferroptosis by removing H3K9 methylation marks, thereby enhancing SLC7A11 expression (210). These insights highlight the critical role of histone methylation in modulating ferroptosis pathways. Additionally, histone ubiquitination, exemplified by H2Bub1, acts as a prerequisite for establishing H3K4me3 and H3K79me3 marks and further fine-tunes ferroptosis through the regulation of SLC7A11 and related gene expression (211).

Nevertheless, current research establishing a direct link between histone modifications and ferroptosis in AD remains limited, with most evidence being indirect. Future investigations should integrate animal models and *in vitro* systems to systematically analyze the dynamic relationship between ferroptosis biomarkers and specific histone modification patterns (e.g., H3K9me3, H3K27me3) across different stages of the disease. Such studies hold significant promise for informing the development of novel therapeutic strategies and providing critical frameworks for treatment efficacy evaluation.

#### 6.4.2 DNA methylation

Aberrant DNA methylation constitutes a significant epigenetic mechanism in neurodegenerative disorders (212). This modification primarily occurs within CpG islands (500–1000 bp regions with >60% C/G content) located in gene promoter regions. Catalyzed by DNA methyltransferases (DNMT1, DNMT2, DNMT3a, DNMT3b), it involves the transfer of a methyl group from S-adenosylmethionine (SAM) to the 5’ carbon position of cytosine residues, subsequently silencing gene expression by impeding transcription factor binding. In AD, age-associated declines in DNMT1 and DNMT3a2 expression contribute to abnormal methylation of memory-related genes (e.g., reelin), triggering cognitive dysfunction (213, 214). Human studies further demonstrate DNMT3a involvement in improving cognitive function in patients with mild cognitive impairment (215). Notably, reduced methylation levels within the APP promoter region promote Aβ generation. Aβ itself can induce global genomic hypomethylation while concurrently promoting hypermethylation of the neprilysin (NEP) promoter, establishing a self-perpetuating pathological cycle (216, 217). GPX4, a critical regulator of ferroptosis, exhibits significantly reduced expression in AD neurons. This observation presents a potential parallel to findings in cancer research, where DNA methylation directly governs GPX4 expression (218). While insights from oncology provide valuable clues for understanding ferroptosis regulation in AD, the distinct pathological microenvironment of AD—characterized by Aβ deposition, tau pathology, and chronic neuroinflammation—suggests that these epigenetic regulatory mechanisms likely exhibit disease-specific features. Consequently, direct investigations within AD models and patient cohorts are essential to validate the precise mechanistic role of DNA methylation in regulating ferroptosis during neurodegeneration.

#### 6.4.3 Non-coding RNA

microRNA (miRNA) are single-stranded non-coding RNAs typically composed of 19–25 nucleotides. These molecules play critical regulatory roles in diverse biological processes, including metabolism, neurodevelopment, synaptic plasticity, cell death, and other neurobiological functions. In the cerebrospinal fluid of AD patients, dysregulation of numerous miRNAs correlates with disease progression. Notably, upregulated expression of miR-9, miR-125b, miR-146a, and miR-155 promotes neuroinflammation by suppressing complement factor H (CFH) expression (219). Additionally, miR-9 contributes to AD pathogenesis by inducing ferroptosis through targeted inhibition of SLC7A11, thereby blocking GSH synthesis, and additionally participates in regulating NFTs formation (220). Similarly, miR-137 activates ferroptosis by downregulating SLC1A5, leading to impaired cysteine uptake and consequent disruption of GSH synthesis (221). Insights from oncology research, demonstrating that miR-522 inhibits lipid ROS accumulation and suppresses ferroptosis by targeting ALOX15, offer valuable perspectives for understanding ferroptosis regulation in AD (222). While these findings establish important connections, direct evidence specifically linking miRNA-mediated regulation to neuronal ferroptosis in AD remains limited. Future research should prioritize elucidating the roles of specific miRNAs in driving ferroptosis within AD neurons and evaluating their therapeutic potential.

### 6.5 Clinical and translational evidence: limitations and prospects

Although preclinical and pathological studies provide substantial support for the role of ferroptosis in Alzheimer’s disease, direct human evidence and clinical translational research remain significantly insufficient, representing a critical bottleneck in current investigations. The main limitations are reflected in the following aspects:

#### 6.5.1 Lack of a specific *in vivo* biomarker system

Currently, no biomarker profile has been established that can specifically and dynamically reflect the ferroptosis process *in vivo*. Although lipid peroxidation products (e.g., 4-HNE, MDA) and systemic iron parameters are altered in bodily fluids of AD patients (e.g., cerebrospinal fluid, blood), these markers lack disease and cell death modality specificity, as similar changes can occur in other neurodegenerative disorders or systemic pathological conditions. Therefore, developing and validating biomarkers that directly indicate neuronal ferroptosis—such as specific oxidized phospholipid profiles, ferroptosis-related proteins (e.g., released HMGB1, modified GPX4), or their metabolic fragments—is crucial for enhancing clinical diagnostic and therapeutic monitoring capabilities.

#### 6.5.2 Immaturity of *in vivo* imaging techniques

Existing neuroimaging technologies can indirectly reflect iron deposition (e.g., QSM) or amyloid burden (e.g., Aβ-PET) but are currently unable to directly and non-invasively visualize the spatiotemporal dynamics of iron-dependent lipid peroxidation, loss of membrane integrity, or ferroptosis-associated mitochondrial morphological changes. Future efforts should focus on developing novel imaging probes or methodologies, such as PET tracers or functional MRI techniques targeting active lipid peroxides, redox imbalance, or ferroptosis-specific membrane damage, to enable quantitative and localized analysis of ferroptosis in the living human brain.

#### 6.5.3 Extremely limited clinical intervention

Most current evidence derives from preclinical models, while interventional clinical trials specifically designed to target the ferroptosis pathway in AD patients remain scarce. Although strategies such as iron chelators (e.g., deferoxamine), antioxidants (e.g., N-acetylcysteine), and GPX4 activation demonstrate neuroprotective potential in animal and cellular models, their safety, tolerability, blood-brain barrier permeability, and ability to modulate disease progression in humans have not been systematically validated. Advancing Phase II/III clinical trials targeting key nodes of ferroptosis (e.g., iron metabolism, GPX4 function, lipid peroxide clearance) is essential to confirm its therapeutic value and translational potential.

In summary, although the ferroptosis hypothesis is mechanistically compelling and supported by evidence from models and post-mortem tissue, its role as a primary therapeutic target in human Alzheimer’s disease remains to be validated. Future research must prioritize the development of specific *in vivo* biomarkers, the advancement of pathophysiology-targeted imaging tools, and the implementation of well-designed clinical trials targeting key nodes of the ferroptosis pathway. Only through such translational efforts can we determine whether modulating ferroptosis represents a viable strategy for delaying or preventing the progression of Alzheimer’s disease.

## 7 Therapeutic strategies and clinical translation

With the deepening understanding of ferroptosis mechanisms, an increasing number of molecular targets have been identified as closely associated with the pathological progression of AD. These key molecules not only provide novel perspectives for elucidating AD pathogenesis but also offer potential targets for developing new neuroprotective agents (223). Currently, targeted neuroprotective drugs acting on ferroptosis pathways are under progressive development, with iron chelators, antioxidants, and free radical scavengers being extensively investigated to achieve neuronal protection through modulation of ferroptosis processes (224). This section summarizes the current research status of reported ferroptosis-related pharmacological agents, analyzing their mechanisms of action and therapeutic effects in AD models, aiming to provide theoretical foundations and structural references for future anti-AD drug design based on ferroptosis mechanisms (**Table 1**).

**Table 1:** Therapeutic strategies targeting ferroptosis and their mechanisms of action.

Category	Compound	Core Mechanism of Action	Efficacy	References
Iron Chelators	DFO	Chelate Fe²⁺ to inhibit the Fenton reaction; Block iron-dependent Aβ fibrillization; Inhibit LOX activity	Reduce hippocampal labile iron concentration; Decrease Aβ plaque burden	[228]
	DFP	Penetrate the BBB and regulate brain iron homeostasis; Chelate iron to inhibit key ferroptosis drivers	May induce neutropenia	[229]
	Curcumin	Potently chelate iron ions; Inhibit oligomer formation; Dissociate pre-existing amyloid plaques	Dissociate existing amyloid plaques in the mouse hippocampus; Inhibit tau hyperphosphorylation in the dentate gyrus	[225]
Amino Acid Derivative	NAC	Promote GSH biosynthesis; Maintain GPX4 activity; Efficiently cross the blood-brain barrier	Inhibit lipid peroxidation, exerting neuroprotective effects	[188]
Natural Iridoid Glycoside	Phillyrin A	Elevate GSH levels; reduce ROS and MDA levels; Activate the Nrf2 signaling pathway; upregulate antioxidant protein expression	Attenuate Aβ-induced neuroinflammation and apoptosis in hippocampal slices; Improve learning and memory in senescence-accelerated mice	[231]
Vitamin K	K1、K3	Exert broad-spectrum ferroptosis suppression; Generate VKH₂	Significant cytoprotection	[86]
Radical-Trapping Antioxidant	Ferrostatin-1 (Fer-1)	Directly trap lipid radicals; Inhibit ferroptosis with superior efficacy	Potent ferroptosis inhibition	[236]
Terpene Lactone	Ginkgolide B	Modulate the Nrf2-dependent pathway; Inhibit PI3K/Akt and TLR4/NF-κB signaling; Induce heme oxygenase-1 expression	Preclinical evidence: Mitigates free radical damage and inflammation, alleviating AD-related cognitive dysfunction	[238]
Fat-Soluble Vitamin	Vitamin E	Potently quenches lipid peroxyl radicals, inhibiting ferroptosis mice	Clinical evidence: Significantly reduced levels in plasma, serum, and CSF of AD patients, inversely correlating with disease severity; Daily supplementation effectively slows cognitive decline in mild-to-moderate AD patients	[246]
PUFAs	ω-3 PUFAs	Influence membrane fluidity and peroxidation susceptibility; Long-term intake reduces CSF 4-HNE levels	Epidemiological evidence: Long-term intake in middle-aged adults lowers preclinical AD risk	[247]
	Eriodictyol	Ameliorates amyloid pathology via the vitamin D receptor-mediated Nrf2/HO-1 signaling pathway	Enhances the amelioration of amyloid pathology	[242]
Natural Compound (Synergizing with Nrf2)	MG53 Protein	Activates the Nrf2 pathway in senescent mesenchymal stem cells	Promotes the development of "sulforaphane-cell" combination therapy	[250]
	7-O-Cinnamoyl-paclitaxel7-O-Feruloyl-paclitaxel	Activate the Nrf2 pathway under oxidative stress, upregulating downstream antioxidant enzymes (HO-1, NQO1)	Alleviate neurotoxicity; Improve short-term memory	[251]

DFO, esferrioxamine; DFP, Deferiprone; LOX, Lipoxygenase; BBB, Blood-Brain Barrier; NAC, N-Acetylcysteine; GSH, Glutathione; GPX4, Glutathione Peroxidase 4; ROS, Reactive Oxygen Species; Nrf2, Nuclear factor erythroid 2-related factor 2; MDA, Malondialdehyde; VKH₂, Vitamin K Hydroquinone; PI3K, Phosphoinositide 3-Kinase; Akt, Protein Kinase B; TLR4, Toll-like Receptor 4; NF-κB, Nuclear Factor kappa-light-chain-enhancer of activated B cells; AD, Alzheimer's Disease; CSF, erebrospinal Fluid; HNE, 4-Hydroxynonenal; HO-1, Heme Oxygenase-1; NQO1, NAD(P)H: quinone oxidoreductase 1;

### 7.1 Iron chelators

#### 7.1.1 Preclinical stage

Natural and synthetic iron chelators in preclinical development exhibit neuroprotective potential by modulating cerebral iron dyshomeostasis, though their translational value requires further validation. The natural polyphenol curcumin provides broader neuroprotection; beyond efficient iron chelation, its phenolic structure directly inserts into the Aβ folding core, inhibiting oligomer formation (225). Animal models demonstrate that curcumin treatment dissociates existing Aβ plaques in the mouse hippocampus and significantly inhibits tau hyperphosphorylation at Ser202/Thr205 in the dentate gyrus (226, 227). However, curcumin faces inherent limitations including poor bioavailability and inefficient BBB penetration, which hinder its clinical translation. Synthetic chelators like desferrioxamine (DFO) have been extensively validated in preclinical models: studies confirm DFO significantly reduces free iron concentrations in the hippocampus of APP/PS1 mice, concurrently decreasing Aβ plaque burden and improving cognitive function (228). Nevertheless, DFO’s clinical applicability is constrained by poor BBB penetration and non-oral administration route, prompting the development of modified delivery systems(e.g., DFO-gold nanoparticle conjugates, NCT05022472) currently under preclinical optimization.

#### 7.1.2 Clinical stage

Deferiprone (DFP) represents a promising clinical-stage iron chelator with oral absorbability and BBB permeability, demonstrating a favorable safety profile with low systemic toxicity, which positions it as a viable strategy for countering iron dyshomeostasis and ferroptosis in AD therapeutics (229). Despite its translational advantages, clinical application of DFP and other iron chelators is not without risks: neutropenia remains a serious adverse event frequently observed in patients receiving iron chelation therapy (230). Additionally, given their systemic impact on iron homeostasis, regular monitoring of iron metabolism and vital organ functions—particularly hepatic and renal systems—is essential to minimize adverse events and ensure therapeutic safety. Ongoing clinical research focuses on optimizing brain-targeted delivery (e.g., NCT05022472, a trial evaluating DFO-gold nanoparticle conjugates) and developing structural analogs (e.g., CNB-001) to enhance efficacy while reducing off-target effects.

### 7.2 Antioxidants

#### 7.2.1 Preclinical stage

Phillyrin A, a naturally occurring iridoid glycoside isolated from Forsythia suspensa, has garnered attention for its broad biological activities including anti-inflammatory, neuroprotective, and ferroptosis-inhibitory effects (231, 232). *In vitro* studies demonstrate its efficacy in mitigating Aβ-induced neuroinflammation and apoptosis in hippocampal slices (233), while HT22 cell models confirm phillyrin A counteracts erastin-induced ferroptosis by elevating GSH content, reducing ROS and malondialdehyde (MDA) levels. Preclinical animal studies further validate its neuroprotective potential: phillyrin A improves learning and memory in senescence-accelerated mice, activates the Nrf2 signaling pathway in APP/PS1 mice, upregulates antioxidant proteins (NQO1, GPX4), and normalizes cerebral iron content through modulation of TFRC, DMT1, FTH, and FTL gene expression (234). Ultrastructural analyses also indicate its ability to mitigate erastin-triggered ferroptotic mitochondrial alterations (e.g., shrinkage and cristae disruption). However, phillyrin A’s clinical translation is currently limited by insufficient data on BBB penetration efficiency, long-term safety, and optimal dosing regimens.

#### 7.2.2 Clinical stage

N-acetylcysteine (NAC), an acetylated derivative of cysteine naturally present in foods like onions, is a clinical-stage antioxidant with well-characterized BBB penetration and redox-modulating effects in the central nervous system. As a direct precursor of GSH, NAC replenishes intracellular cysteine pools to enhance GSH biosynthesis and bolster cellular antioxidant defenses (235). In AD, NAC elevates GSH levels to sustain GPX4 activity, thereby reducing lipid peroxidation accumulation and inhibiting key ferroptosis pathways for neuroprotection (188).

Vitamin K compounds (phylloquinone, menaquinone-4/MK4, menadione) exhibit broad-spectrum ferroptosis suppression via a non-canonical FSP1-mediated pathway, identifying them as critical substrates for an endogenous anti-ferroptotic system independent of GSH/GPX4 (86). Nonetheless, natural vitamin K forms (e.g., phylloquinone) achieve brain concentrations only ~2% of plasma levels, substantially below the threshold required for neuronal ferroptosis inhibition. Current clinical advancement strategies include structural engineering of vitamin K analogs (e.g., C11-alkyl chain-modified MK4-C11) to enhance brain bioavailability and adeno-associated virus (AAV)-mediated FSP1 gene delivery (NCT05543659) to restore vitamin K reduction capacity, driving the transformation of vitamin K from a fundamental nutrient into a clinically viable targeted ferroptosis therapeutic.

### 7.3 Free radical scavengers

#### 7.3.1 Preclinical stage

Ferrostatin-1 (Fer-1), a canonical radical-trapping antioxidant (RTA), is a cornerstone of ferroptosis research with robust preclinical efficacy (236). As a first-generation ferroptosis inhibitor, its core mechanism involves direct interception of lipid radicals to terminate peroxidation chain reactions: its arylamine group (-NH-) donates hydrogen atoms (H•) to reduce lipid peroxyl radicals (LOO• → LOOH), a mechanism analogous to vitamin E but with superior ferroptosis-inhibitory efficacy (237). Additionally, Fer-1 reduces lipid peroxidation by diminishing Fe²^+^-mediated oxygen radical levels, mitigating oxidation of polyunsaturated fatty acids (PUFAs) in cellular membranes to preserve integrity. Operating independently of GPX4 or FSP1, Fer-1 exhibits efficacy across diverse ferroptosis models (e.g., RSL3- or erastin-induced ferroptosis). However, its clinical applicability is severely constrained by poor stability and susceptibility to oxidation, limiting its translational potential.

Ginkgolide B, a core bioactive component of Ginkgo biloba extract, demonstrates preclinical therapeutic potential in AD by ameliorating radical damage and inflammation, and exerting anti-ferroptotic effects via modulation of Nrf2-dependent pathways (238–240). Its neuroprotective mechanisms encompass suppression of PI3K/Akt and TLR4/NF-κB signaling, induction of heme oxygenase-1 (HO-1) expression, and regulation of anti-apoptotic/pro-apoptotic protein balance (241), suggesting it alleviates AD-associated cognitive impairment through ferroptosis inhibition (242). Despite these preclinical benefits, ginkgolide B’s clinical translation requires further validation of BBB penetration efficiency and long-term safety.

Edaravone (EDA), another radical scavenger, has recently shown preclinical potential for treating neurodegenerative diseases including AD through modulation of ferroptosis-related pathways (243, 244). Preclinical studies support its neuroprotective effects in AD models, though clinical validation remains pending (245).

#### 7.3.2 Clinical stage

Vitamin E (α-tocopherol) is a clinically validated free radical scavenger with well-established ferroptosis-inhibitory effects: its chromanol phenolic hydroxyl group potently quenches lipid peroxyl radicals (LOO•) to terminate peroxidation chains. Clinical biochemistry studies reveal significantly reduced vitamin E levels in plasma, serum, and cerebrospinal fluid of AD patients, inversely correlating with disease severity. Randomized controlled trials confirm that daily vitamin E supplementation effectively slows cognitive decline in mild-to-moderate AD patients, supporting its role as a disease-modifying agent (246). Beyond vitamin E, nutritional interventions with ω-3 PUFAs have shown clinical relevance for AD prevention: long-term ω-3 PUFA intake in middle-aged adults lowers cerebrospinal 4-HNE levels and reduces preclinical AD risk, providing scientific rationale for nutrition-based primary prevention strategies targeting ferroptosis (247).

### 7.4 Synergistic intervention

#### 7.4.1 Combination of Nrf2 activators and Aβ clearance

Nrf2 activators centered on sulforaphane are driving a paradigm shift in AD treatment from traditional single-pathway approaches toward a dual-track strategy targeting both pathological clearance and ferroptosis blockade. Nuclear-translocated Nrf2 directly downregulates BACE1 and its antisense RNA (BACE1-AS), suppressing Aβ generation; concurrently, it coordinately upregulates GPX4 expression, enhances GSH synthesis, and modulates iron metabolism proteins to effectively inhibit neuronal ferroptosis (248, 249). Natural compounds eriodictyol and ginkgolide B potentiate this effect: eriodictyol ameliorates amyloid pathology via vitamin D receptor (VDR)-mediated Nrf2/HO-1 signaling, while ginkgolide B reinforces the Nrf2/GPX4 axis to mitigate neuroinflammation-induced ferroptosis (242). Additionally, MG53 protein activates the Nrf2 pathway in senescent human umbilical cord mesenchymal stem cells (hUC-MSCs), significantly enhancing their capacity to clear Aβ deposits and suppress neuronal ferroptosis, thereby advancing sulforaphane-cell combination therapy (250).

Two hybrid compounds derived from natural products—7-O-cinnamoyl-paclitaxel and 7-O-feruloyl-paclitaxel—exhibit potent synergistic neuroprotection in HT22 cell injury models (251). In AD mice induced by intracerebral oligomeric Aβ25–35 peptide injection, both compounds individually alleviate neurotoxicity and improve short-term memory (validated by novel object recognition and Morris water maze tests), likely through Nrf2 pathway activation under oxidative stress. This upregulates downstream antioxidant enzymes (e.g., HO-1, NQO1) and maintains intracellular GSH levels, reducing ROS accumulation and enhancing neuronal resistance to Aβ toxicity (251).

Despite the promising preclinical therapeutic potential, clinical translation faces multiple challenges: sustained Nrf2 activation regulates over 200 target genes, posing oncogenic risks (e.g., pancreatic ductal hyperplasia); natural Nrf2 activators typically exhibit suboptimal blood-brain barrier penetration; and late-stage AD neuronal apoptosis substantially diminishes Nrf2 activation efficiency. Current research focuses on overcoming these limitations through nanotechnology-enabled brain-targeted delivery systems and real-time monitoring techniques, aiming to accelerate the clinical application of Nrf2-based combination therapies.

#### 7.4.2 Combined treatment with metformin and alpha-lipoic acid

Lipoic acid, a potent antioxidant, activates the cytoprotective Nrf2 pathway. Metformin may suppress the key ferroptosis driver ACSL4 (which promotes toxic lipid biosynthesis) through AMPK pathway activation or reduce iron-dependent cellular damage by modulating iron metabolism-related proteins (e.g., downregulating transferrin receptor). Their combination theoretically offers a dual ferroptosis-inhibitory mechanism via concurrent Nrf2 activation and AMPK/ACSL4 suppression, particularly relevant in neurodegenerative contexts like AD.

Interim results from the NCT04886001 trial demonstrated clinical efficacy of the combination: it significantly improved cognitive function in early AD patients (assessed by ADAS-Cog13, with enhanced effects in APOE4-negative subgroups) and reduced CSF p-tau pathology compared with placebo, alongside an acceptable safety profile. However, the trial had limitations: it neither measured ferroptosis biomarkers (e.g., ACSL4/Ptgs2) nor included monotherapy control arms, thereby precluding validation of the putative anti-ferroptotic mechanism or synergistic superiority of the combination. This mechanistic hypothesis requires confirmation through dedicated studies.

#### 7.4.3 Multi-target cocktail approach

Current therapeutic strategies for AD prioritize pharmacotherapy supplemented by rehabilitative training, as single-target or monotherapeutic approaches yield largely unsatisfactory outcomes, establishing multitarget combination therapies (“cocktail therapy”) as the emerging mainstream paradigm. This integrates multiple clinical mechanisms—including neuroprotection, targeted modulation, and risk factor intervention—to comprehensively address core AD pathologies: Aβ deposition, tau hyperphosphorylation, ferroptosis, and neuroinflammatory cross-talk.

The regimen emphasizes stage-specific interventions: early-stage management combines Aβ-clearing agents (e.g., aducanumab) with ferroptosis inhibitors (e.g., sulforaphane via Nrf2/GPX4 activation) to reduce Aβ plaques and counteract oxidative stress/lipid peroxidation; mid-stage progression incorporates tau-targeting and anti-inflammatory compounds such as phillyrin A to mitigate chronic neuroinflammation (252). late-stage maintenance therapy introduces human umbilical cord mesenchymal stem cell (hUC-MSC) transplantation to promote neuronal regeneration.

To ensure effective and stable intracerebral drug concentrations, continuous optimization of delivery systems is pursued, exemplified by hyperpermeable selenium nanoparticles coupled with focused ultrasound for precise antibody targeting. Focused ultrasound-microbubble technology represents a promising non-invasive delivery approach, offering minimal invasiveness and enhanced safety for CNS drug administration. Further advancing this field, the clinical trial NCT05218903 is evaluating subcutaneous versus intravenous delivery of lecanemab in early Alzheimer’s disease. The study assesses the safety, tolerability, and bioactivity of the subcutaneous formulation; its primary goal is to determine whether this route maintains therapeutic efficacy while offering greater convenience. Results are expected in 2025.

### 7.5 Critical discussion of translational challenges

The translation of ferroptosis-targeting agents for AD faces multiple interconnected challenges, with BBB penetration, off-target effects, long-term safety, and perturbations of iron metabolism and immune function being the most prominent. BBB penetration remains a primary bottleneck: natural compounds (curcumin, phillyrin A) and some synthetic agents (DFO) exhibit poor brain bioavailability, necessitating delivery system modification (e.g., nanoparticle conjugates) or structural optimization (e.g., vitamin K analogs). While agents like DFP and NAC demonstrate favorable BBB penetration, their systemic effects on iron metabolism raise concerns: iron chelators can induce unintended iron deficiency in peripheral tissues, while antioxidants may disrupt physiological redox balance, highlighting the need for brain-targeted delivery to minimize off-target effects.

Long-term safety profiles are another critical consideration. Neutropenia associated with iron chelation therapy and potential hepatic/renal toxicity underscore the importance of continuous monitoring of organ function during clinical application. For nutritional antioxidants (vitamin E, ω-3 PUFAs), long-term high-dose supplementation may carry risks (e.g., increased bleeding risk with vitamin E), requiring careful dose optimization. Additionally, the interplay between ferroptosis inhibition and immune function is poorly understood: iron is essential for immune cell activation, and systemic iron chelation could compromise immune surveillance, representing an understudied off-target effect.

Future translational efforts must prioritize strategies that enhance brain specificity (e.g., targeted delivery systems, BBB-penetrating analogs) while conducting comprehensive long-term safety assessments. Integrating multi-mechanistic agents (e.g., curcumin’s dual iron chelation and Aβ inhibition, CNB-001’s synergistic antioxidant and anti-inflammatory effects) may improve efficacy while reducing required doses, thereby minimizing off-target risks. Finally, rigorous clinical trials evaluating not only cognitive outcomes but also biomarkers of iron metabolism, lipid peroxidation, and immune function will be essential to validate the safety and efficacy of ferroptosis-targeting therapies for AD.

## 8 Limitations

In the context where AD research has encountered therapeutic bottlenecks with traditional “Aβ/tau” targets, the introduction of ferroptosis offers a novel and crucial perspective for understanding the disease. It not only molecularly links oxidative stress and iron dyshomeostasis with classical protein pathologies, forming an integrated pathogenic framework of “iron-Aβ/tau-neuronal death,” but also reveals an actionable cell death execution pathway. This provides substantial hope for developing disease-modifying therapies that go beyond mere protein clearance.

However, as this promising field transitions from basic discovery to clinical application, it faces a series of core challenges and limitations that must be addressed. Current evidence predominantly shows correlative phenomena such as iron accumulation, lipid peroxidation, and GPX4 downregulation. Yet, the precise spatiotemporal causal sequence among these three elements remains unconfirmed by longitudinal human studies. A contributory unanswered question is: is ferroptosis an early key driver of neurodegeneration, or a late-stage “executor” triggered by other pathologies?

Significant shortfalls also exist in clinical translation. While QSM-based MRI can non-invasively quantify brain iron, highly specific and sensitive fluid-based (blood/CSF) ferroptosis biomarkers are still lacking. Existing markers (e.g., 4-HNE, MDA) generally lack disease specificity, and their integrative analysis with classical AD biomarkers like Aβ and tau is insufficient. Regarding therapeutic strategies, interventions based on animal models (e.g., iron chelators, antioxidants) face a dual dilemma of target specificity and safety. Systemic drug administration struggles to precisely target affected brain regions and easily disrupts systemic iron homeostasis. Long-term inhibition of core pathways like GPX4 carries unknown risks, while targeting upstream regulators like Nrf2 harbors potential off-target effects due to their broad biological functions. Furthermore, mechanistic research heavily relies on transgenic AD mouse models (e.g., APP/PS1), which primarily model Aβ pathology. The similarity of their iron dysregulation and ferroptosis phenotypes to human AD remains questionable, limiting the translational power of the conclusions.

Ferroptosis does not occur in isolation; it is intricately interlinked with other core pathological processes in AD, including neuroinflammation and mitochondrial dysfunction. To advance this field, ferroptosis must be positioned within a broader systems biology framework of AD. Elucidating its precise mechanistic contribution and hierarchical significance within this complex pathogenic network is essential for achieving fundamental theoretical insights and enabling successful clinical translation.

## 9 Conclusion

AD is a neurodegenerative disorder characterized by neuronal loss and cognitive dysfunction, featuring hallmark pathological lesions including Aβ plaques and NFTs resulting from aberrant tau hyperphosphorylation. Although neuronal demise involves crosstalk among multiple cell death pathways, the contribution of ferroptosis has become increasingly evident. Ferroptosis—an iron-dependent regulated cell death distinct from apoptosis, necrosis, and autophagy—plays an important role in AD pathogenesis. Its core mechanisms encompass imbalance in iron homeostasis, enhanced lipid peroxidation, and GSH metabolic dysregulation, intersecting fundamentally with AD pathophysiology. Inhibiting ferroptosis via iron chelators, radical-trapping agents, and other interventions demonstrates significant improvements in neuronal survival and synaptic function across diverse preclinical models, establishing ferroptosis targeting as a promising novel therapeutic strategy for AD.

The current AD therapeutic landscape faces substantial challenges, with the lack of effective disease-modifying drugs underscoring the urgent need for preventive interventions. While healthy lifestyle modifications may partially mitigate age-related cognitive decline, precise molecular targeting remains indispensable. The efficacy of ferroptosis inhibitors in AD models reveals three key therapeutic development avenues: modulation of iron metabolism, suppression of lipid peroxidation, and restoration of GSH system functionality. Future research should prioritize elucidating dynamic iron transport mechanisms across the blood-brain barrier, validating combination therapies targeting synergistic ferroptosis regulators (e.g., Nrf2/GPX4/FSP1), and advancing preclinical findings toward biomarker identification and precision intervention protocols. In summary, as a critical component in AD pathology, mechanistic investigation and therapeutic exploration of ferroptosis hold significant importance, necessitating further multidimensional and interdisciplinary research to comprehensively understand its relationship with AD and drive innovative treatment development.
